# Analysis of a degron-containing reporter protein GFP-CL1 reveals a role for SUMO1 in cytosolic protein quality control

**DOI:** 10.1016/j.jbc.2022.102851

**Published:** 2022-12-29

**Authors:** Wei Wang, Jian Lu, Wei-Chih Yang, Eric D. Spear, Susan Michaelis, Michael J. Matunis

**Affiliations:** 1 Department of Biochemistry and Molecular Biology, Johns Hopkins University, Bloomberg School of Public Health, Baltimore, Maryland, USA; 2 Department of Cell Biology, Johns Hopkins University, School of Medicine, Baltimore, Maryland, USA

**Keywords:** small ubiquitin-like modifier, ubiquitin-proteasome system (UPS), protein quality control, CL1 degron, Cdc48/p97, AZC, azetidine-2-carboxylic acid, ER, endoplasmic reticulum, ERAD, endoplasmic reticulum–associated degradation, MBP, maltose-binding protein, NES, nuclear export signal, NLS, nuclear localization signal, PQC, protein quality control, SIM, SUMO-interacting motif, STUbL, SUMO-targeted ubiquitin ligase, SUMO, small ubiquitin-related modifier, TA, tail-anchored, TMD, transmembrane domain, Ufd1, ubiquitin fusion degradation protein 1

## Abstract

Misfolded proteins are recognized and degraded through protein quality control (PQC) pathways, which are essential for maintaining proteostasis and normal cellular functions. Defects in PQC can result in disease, including cancer, cardiovascular disease, and neurodegeneration. The small ubiquitin-related modifiers (SUMOs) were previously implicated in the degradation of nuclear misfolded proteins, but their functions in cytoplasmic PQC are unclear. Here, in a systematic screen of SUMO protein mutations in the budding yeast *Saccharomyces cerevisiae*, we identified a mutant allele (*Smt3-K38A/K40A)* that sensitizes cells to proteotoxic stress induced by amino acid analogs. *Smt3-K38A/K40A* mutant strains also exhibited a defect in the turnover of a soluble PQC model substrate containing the CL1 degron (NES-GFP-Ura3-CL1) localized in the cytoplasm, but not the nucleus. Using human U2OS SUMO1- and SUMO2-KO cell lines, we observed a similar SUMO-dependent pathway for degradation of the mammalian degron-containing PQC reporter protein, GFP-CL1, also only in the cytoplasm but not the nucleus. Moreover, we found that turnover of GFP-CL1 in the cytoplasm was uniquely dependent on SUMO1 but not the SUMO2 paralogue. Additionally, we showed that turnover of GFP-CL1 in the cytoplasm is dependent on the AAA-ATPase, Cdc48/p97. Cellular fractionation studies and analysis of a SUMO1-GFP-CL1 fusion protein revealed that SUMO1 promotes cytoplasmic misfolded protein degradation by maintaining substrate solubility. Collectively, our findings reveal a conserved and previously unrecognized role for SUMO1 in regulating cytoplasmic PQC and provide valuable insights into the roles of sumoylation in PQC-associated diseases.

Protein quality control (PQC) maintains proteostasis in cells by monitoring proper folding of native proteins and removing damaged or misfolded proteins through multiple cellular degradation pathways. Compromised PQC can result in aggregation of cytotoxic aberrant proteins, which are associated with numerous human diseases, including cancer, cardiovascular disease, and neurodegeneration (1, 2, 3). Modulating PQC function therefore represents a potential therapeutic strategy to prevent and treat these diseases, but to do so demands a more comprehensive understanding of the relevant regulatory mechanisms. Eukaryotic cells evolved compartmentalized and interconnected PQC machineries, with endoplasmic reticulum–associated degradation (ERAD) being the best characterized (4). In comparison, far less is known about the mechanisms that regulate cytoplasmic PQC, in part due to its intrinsic complexity.

Emerging evidence indicates that the small ubiquitin-related modifiers (SUMOs) play critical roles in PQC (5, 6, 7). For example, sumoylation affects aggregation of multiple proteins associated with neurodegeneration (8, 9). Recent studies also revealed a role for sumoylation in maintaining proteostasis in cardiomyocytes (10, 11). SUMOs are conjugated to proteins *via* a three-step enzymatic cascade that is similar to ubiquitylation, but they have functions distinct from ubiquitin (12). In lower eukaryotes such as *Saccharomyces cerevisiae*, a single SUMO protein, Smt3, is expressed, whereas vertebrates express five SUMO paralogues, with SUMO1, SUMO2, and SUMO3 being the best characterized (13). SUMO2 and SUMO3 share ∼97% similarity in their amino acid sequence and are commonly referred to as SUMO2/3 but share only ∼50% sequence identity to SUMO1. SUMO2 and SUMO3 form polymeric chains through a conserved lysine (K11) embedded within a consensus modification site at the N-terminus (14, 15). In contrast, SUMO1 lacks consensus modification sites and forms polymeric chains less efficiently and may also serve as a terminator of poly-SUMO2/3 chains (16, 17). SUMO1 and SUMO2/3 also interact with distinct subsets of proteins through noncovalent association with SUMO-interacting motifs (SIMs) in these proteins (18). These unique features give SUMO1 and SUMO2/3 the potential to play distinct and nonredundant roles in regulating cellular processes, although a direct comparative analysis of SUMO paralogue functions in PQC has yet to emerge.

Sumoylation and the ubiquitin-proteasomal degradation pathways are bridged by SUMO-targeted ubiquitin ligases (STUbLs), ubiquitin E3 ligases that recognize poly-SUMO chains (19). For example, SUMO2/3 participates in a nuclear PQC pathway that involves poly-SUMO2/3 modification of misfolded proteins and subsequent recognition and ubiquitylation of these proteins by the STUbL protein RING-finger protein 4 (RNF4) (7). Recently, a second STUbL protein, RNF111 (Arkadia), was found to preferentially target proteins carrying SUMO1-capped SUMO2/3 hybrid conjugates for proteasomal degradation (17). However, roles for SUMOs in cytoplasmic PQC, especially that of SUMO1, have so far not been reported.

In the current study, we identified a conserved SUMO-dependent pathway for degradation of cytosolic PQC model substrates containing the CL1 degron (NES-GFP-Ura3-CL1 in yeast and NES-GFP-CL1 (NESGFPu) in human U2OS cells) but not for their nuclear-localized counterparts. The CL1 degron consists of a 16 amino acid degradation signal which forms a destabilizing amphipathic α-helix and triggers rapid proteasomal degradation. The CL1 degron was originally identified in a *S. cerevisiae* screen for degradation signals that destabilize the cytosolic protein Ura3 and depend on the Ubc6-Ubc7 ubiquitin E2-conjugating enzymes (20). Subsequent studies revealed that degradation of CL1-containing proteins in yeast requires targeting to the cytosolic face of the endoplasmic reticulum (ER) membrane where they are recognized and ubiquitylated by the ER-resident ubiquitin E3 ligase, Doa10 (21, 22). Studies also showed that recognition and degradation is dependent on two protein chaperones, Ssa1 and Ydj1, and the AAA-ATPase, Cdc48 (21). Studies of CL1 fusion proteins in mammalian cells further revealed that recognition of the CL1 degron as a ubiquitin-dependent degradation signal is conserved from yeast to humans (23, 24). Consistent with this, recognition and turnover of CL1-fusion proteins in human cells involves two ER-resident E3 ligases, MARCH6 (homologous to yeast Doa10) and TRC8 (unique to humans) (24).

In this study, we show that SUMO1 promotes GFP-CL1 degradation in human cells upstream of the ubiquitin-proteasome machinery by improving solubility and enhancing downstream modification by ER-localized ubiquitin E2-E3 machinery. We also show that, as in yeast, the AAA-ATPase VCP/p97 (homologous to Cdc48) is required for efficient GFP-CL1 turnover and functions in the same pathway with SUMO1. Results from our studies reveal a novel mechanism by which SUMO1 impacts cytoplasmic PQC, which complements previous knowledge of poly-SUMO2/3–mediated nuclear PQC.

## Results

### Identification of an yeast Smt3 mutant strain sensitive to amino acid analogs

**Figure 1:**
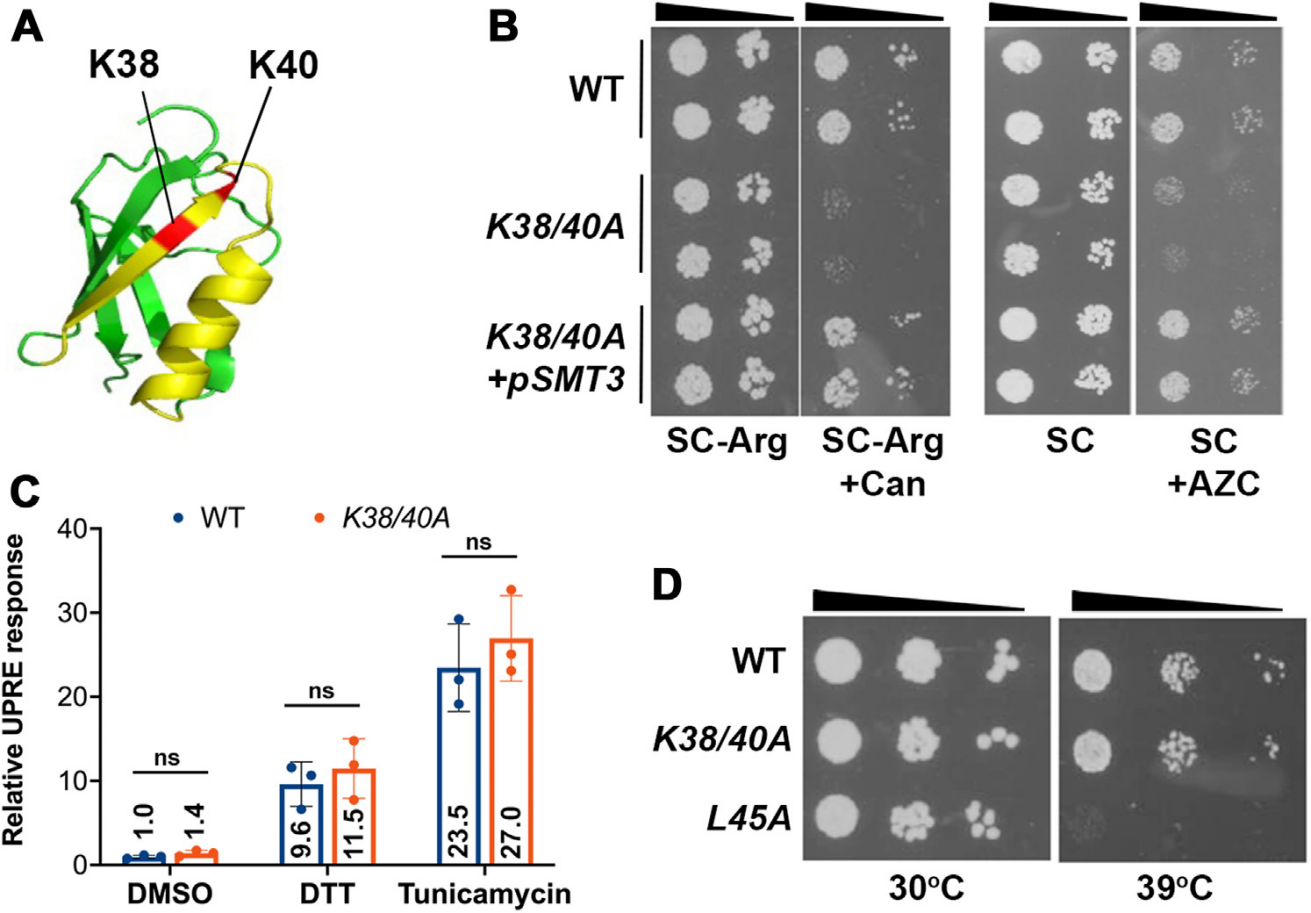
**The yeast *Smt3-K38/40A* mutant strain is sensitive to amino acid analogs.***A*, lysines K38 and K40 are located in the β strand of the SIM-binding surface (highlighted in *yellow*) of the Smt3 structure (PDB: 1EUV). *B*, the *Smt3-K38/40A* strain is sensitive to canavanine (Can) and azetidine-2-carboxylic acid (AZC), and the sensitivities are complemented by WT *SMT3*. Ten-fold serial dilutions were used. *C*, the unfolded protein response (UPR) is appropriately upregulated upon ER stress in the *Smt3-K38/40A* strain. Beta-Gal activity was measured in WT or *Smt3-K38/40A* strains carrying the 4XUPRE-driven lacZ reporter plasmid after treatment with DTT (2 mM), tunicamycin (1 mg/ml), or DMSO for 2 h. The relative value for DMSO-treated WT sample was set to 1. Error bars represent SDs from three biological replicates. Statistical analysis was performed using an unpaired *t* test. *p* = 0.135 (DMSO), 0.507 (DTT), 0.453 (Tunicamycin), respectively. *D*, the *Smt3-K38/40A* strain is not temperature-sensitive. *Smt3-L45A* was used as a positive control. Ten-fold serial dilutions were used. SIM, SUMO-interacting motif; ER, endoplasmic reticulum.

We previously developed a library of >250 mutant alleles of the single *S. cerevisiae* SUMO gene, *SMT3*, coding for proteins with single or multiple amino acid substitutions or deletions (25). Forty-five of these alleles exhibited conditional growth defects under a variety of stress conditions, including in the presence of DNA-damaging agents and under conditions that induce protein misfolding. Of particular interest were a subset of alleles coding for mutations on the surface of Smt3 predicted to affect interactions with downstream effector proteins containing SIMs. These included K38E and K40E mutations which exhibited selective growth defects in the presence of DNA-damaging agents hydroxyurea and methyl methanesulfonate (25). In the course of further characterizing these specific mutant strains, we developed a K38/40A double mutant strain and observed additional growth defects in the presence of canavanine and azetidine-2-carboxylic acid (AZC), which are amino acid analogs that induce protein misfolding when incorporated into newly synthesized polypeptides (Fig. 1, *A* and *B*) (26). Specifically, yeast expressing the *Smt3-K38/40A* allele exhibited severe growth defects compared to the WT strain when cultured on plates containing 1 μg/ml canavanine or 1 μM AZC. Importantly, the observed growth defects could be rescued by the expression of WT *SMT3* (Fig. 1*B*).

To assess sensitivity of the K38/40A mutant strain to protein misfolding in the ER, we also treated cells with known ER stress inducers DTT and tunicamycin and found that activation of the unfolded protein response was not affected in the *Smt3-*K38/40A strain (Fig. 1*C*) (27). Similarly, the *Smt3-K38/40A* strain showed no appreciable growth defects compared to WT strain when cultured at high temperature (39 °C), in contrast to the *Smt3-L45A* strain which we previously identified as heat-sensitive (Fig. 1*D*) (25). Thus, the *Smt3-K38/40A* strain exhibits a selective sensitivity to protein misfolding caused by incorporation of the amino acid analogs canavanine and AZC.

**Figure 2:**
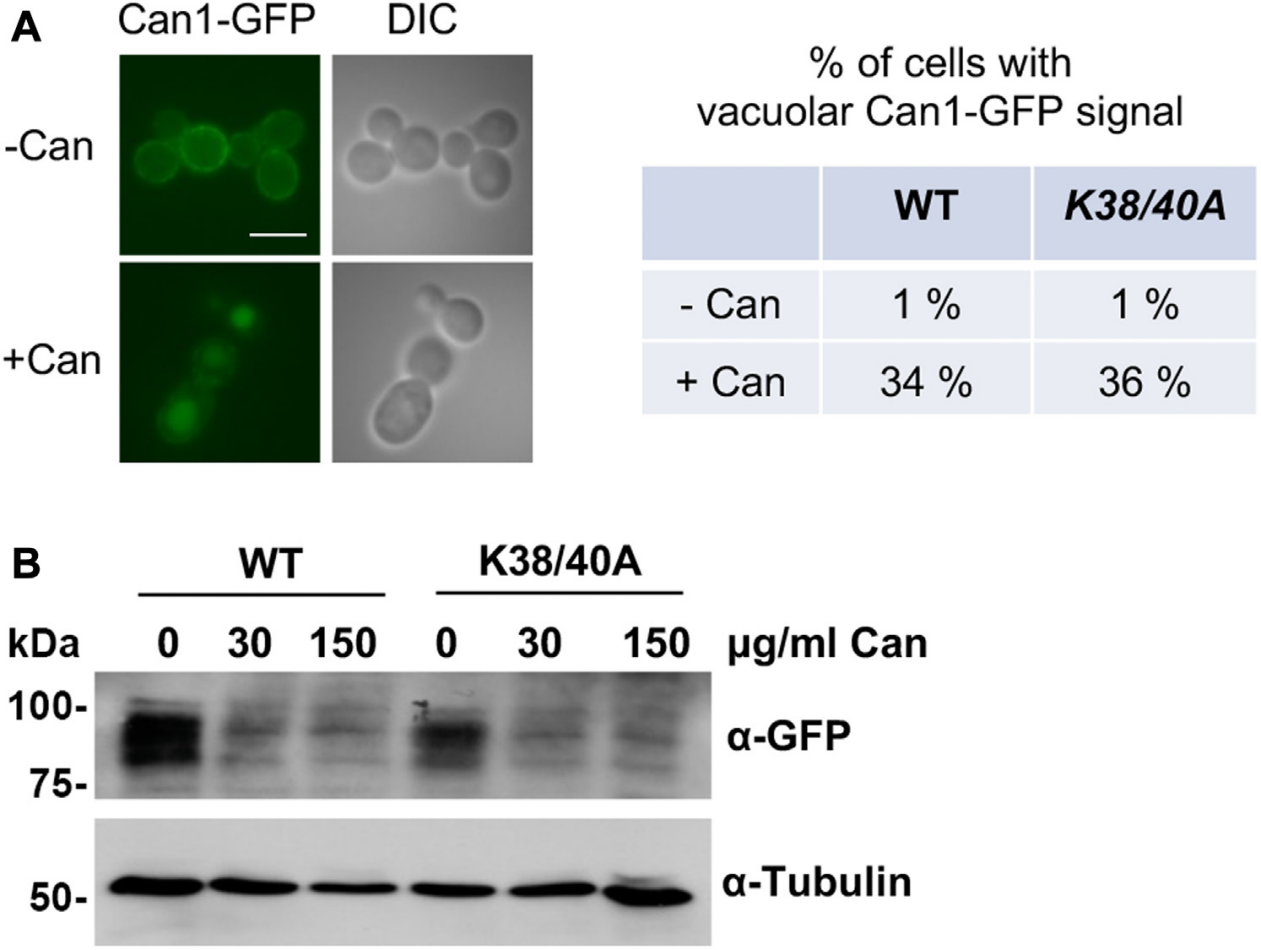
**Can1 localization and levels are appropriately regulated following canavanine treatment in the *Smt3-K38/40A* strain.***A*, log-phase WT or *Smt3-K38/40A* strains were treated with canavanine (150 mg/ml) or untreated for 2 h. Can1-GFP localization was determined by fluorescence microscopy and localization to vacuoles was quantified (n = 200 cells). Scale bar represents 5 μm. *B*, log-phase WT or *Smt3-K38/40A* strains were treated with the indicated concentrations of canavanine for 2 h. Downregulation of Can1-GFP expression was assessed by immunoblot analysis with anti-GFP antibodies. Tubulin was detected as a loading control.

The amino acid permeases that mediate AZC and canavanine import, Gap1 and Can1 respectively, undergo substrate-induced downregulation through ubiquitin-mediated endocytosis (28). To investigate whether the sensitivity to canavanine may be caused by a defect in Can1 internalization and downregulation, we characterized Can1-GFP localization and vacuolar turnover following canavanine treatment. We observed normal localization of Can1 to vacuoles and turnover in the *Smt3-K38/40A* strain, indicating that sensitivity to canavanine is not due to defects in Can1 downregulation (Fig. 2, *A* and *B*).

### The Smt3-K38/40A strain is defective in turnover of a cytosolic PQC reporter protein

**Figure 3:**
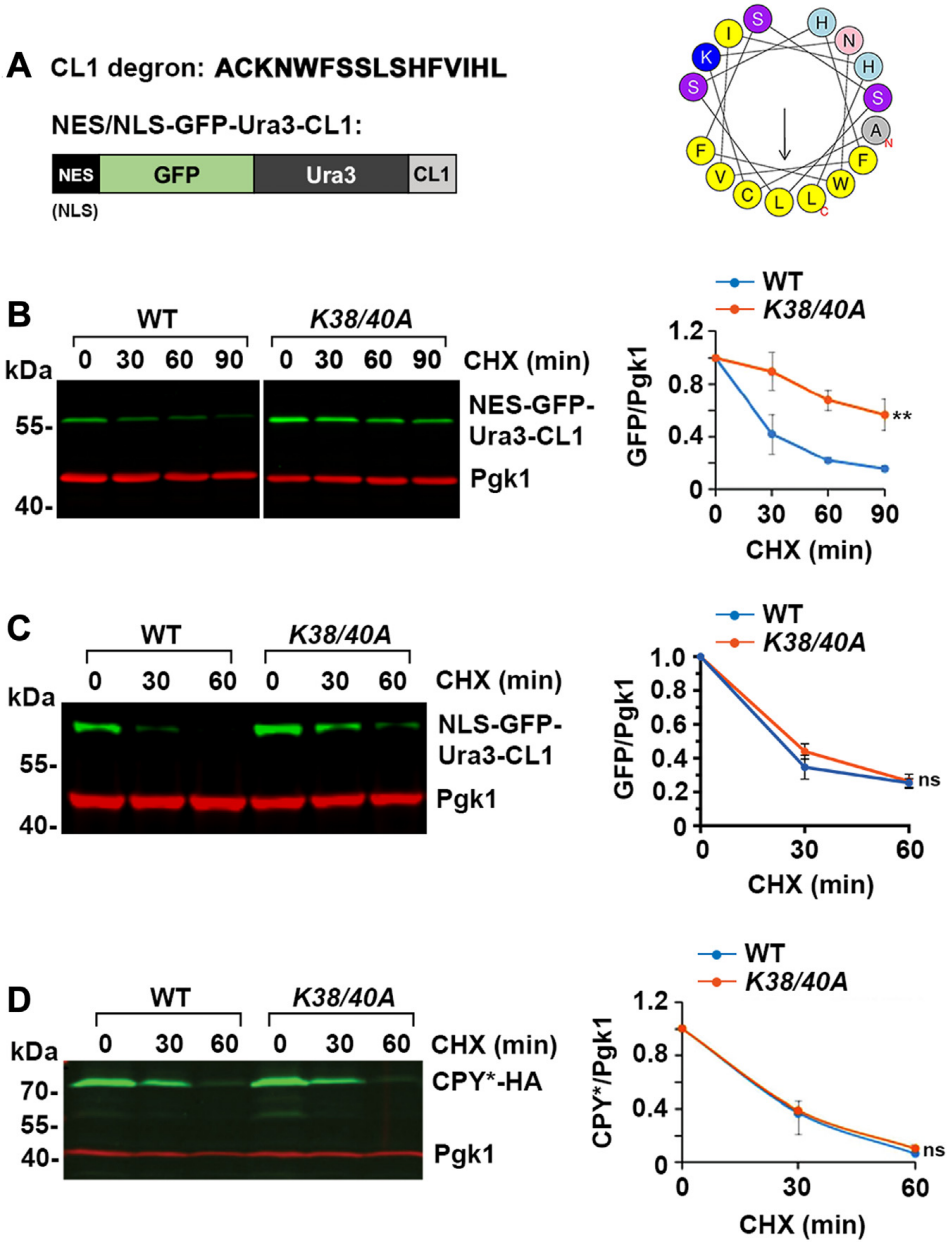
**The *Smt3-K38/40A* mutant strain exhibits specific defects in the degradation of a cytosolic CL1degron–containing reporter protein.***A*, diagram of the 16 amino acid CL1 degron, predicted amphipathic ⍺-helical structure, and schematic of GFP-Ura3-CL1 proteins. CL1 helical structure was predicted using the HeliQuest online prediction tool. *B*, analysis of cytosolic reporter protein NES-GFP-Ura3-CL1 revealed defective degradation in the *Smt3-K38/40A* strain compared to WT strain. Reporter protein expression was induced with galactose and new protein synthesis was inhibited with cycloheximide (CHX) treatment for the indicated times. Total cell lysates were analyzed by immunoblotting with GFP-specific antibodies. Pgk1 was detected as a loading control. Signals were quantified using ImageJ and the ratio of GFP/Pgk1 was plotted and set to 100% at time 0. Protein half-life was estimated based on the curve where remaining GFP/Pgk1 levels were reduced to 50% of starting levels. Error bars represent the SD from three biological replicates. Statistical analysis was performed on the last time point using unpaired *t* test. ∗∗*p* = 0.002. *C*, stability of nucleus-localized reporter protein NLS-GFP-Ura3-CL1 was not affected in the *Smt3-K38/40A* strain compared to WT strain. Analysis was performed as in B. ns, *p* = 0.748. *D*, stability of ERAD model substrate CPY^∗^-HA was comparable in WT and *Smt3-K38/40A* strains. Analysis was performed as in B. ns, *p* = 0.662. ERAD, endoplasmic reticulum–associated degradation; NES, nuclear export signal; NLS, nuclear localization signal.

To investigate whether one or more PQC pathways may be affected in the *Smt3-K38/40A* strain, we evaluated the expression and half-life of a variety of representative PQC reporter proteins normally targeted for ubiquitin-mediated degradation in the nucleus, at the ER or in the cytosol. We first characterized the stability of a soluble, cytosolic reporter protein, NES-GFP-Ura3-CL1 (where NES is a nuclear export signal), whose ubiquitin-dependent degradation in the cytoplasm is mediated by the ER-associated E3 ligase, Doa10 (Fig. 3*A*) (21). Possible changes in protein stability were assessed by immunoblot analysis of cell lysates prepared following cycloheximide treatment, to inhibit new protein synthesis, for varying lengths of time. We found that NES-Ura3-GFP-CL1 was markedly stabilized in the *Smt3-K38/40A* strain (t_1/2_ > 90 min) compared to WT strain (t_1/2_ < 30 min) (Fig. 3*B*). We also targeted GFP-Ura3-CL1 to the nucleus by addition of a nuclear localization signal (NLS) and found that the degradation of this fusion protein was not affected in the *Smt3-K38/40A* strain (Fig. 3*C*). We also examined the stability of the prototype ERAD reporter protein, CPY^∗^, a mutated variant of carboxypeptidase Y whose extraction from the ER lumen and degradation is dependent on the Hrd1 ubiquitin E3 ligase (29). Our analysis revealed that CPY^∗^ stability was unaffected in the *Smt3-K38/40A* strain compared to WT (t_1/2_ = 20 min) (Fig. 3*D*). Collectively, our findings reveal a novel and selective role for sumoylation in facilitating the ubiquitin-dependent turnover of a soluble, cytosolic PQC reporter protein.

### The Smt3-K38/40A mutant protein is defective in SIM binding

**Figure 4:**
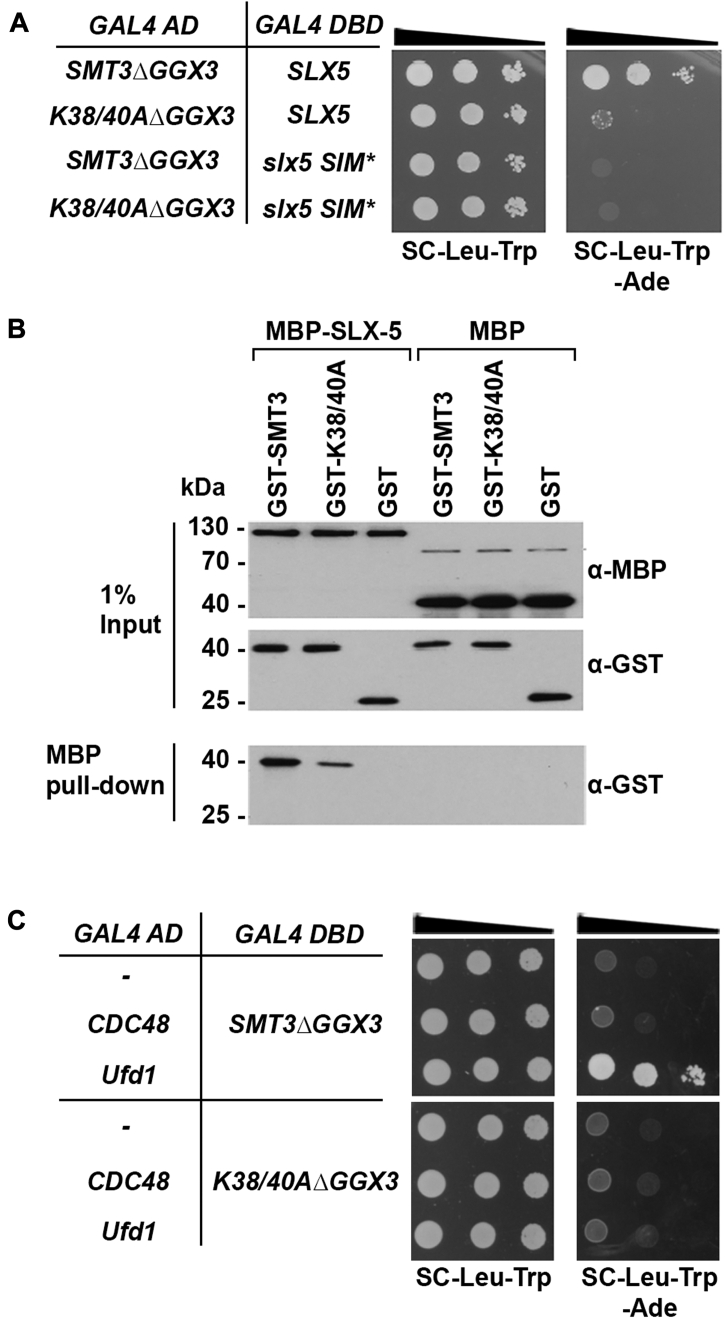
**The Smt3-K38/40A mutant protein is defective in SIM binding.***A*, yeast two-hybrid assays revealed reduced binding between Smt3-K38/40A and Slx5. *Saccharomyces cerevisiae* strains expressing three tandem repeats of Smt3ΔGG or Smt3-K38/40AΔGG fused to Gal4 DNA activation domain (AD), together with strains expressing Slx5 or Slx5-SIM mutant (SIM^∗^) fused to the Gal4 DNA-binding domain (DBD), were generated. Ten-fold serial dilutions of indicated strains were spotted on the indicated media. Protein interactions result in activation of the ADE2 reporter gene and growth on plates lacking adenine (-Ade). *B*, *in vitro* analysis revealed reduced Slx5 binding to Smt3-K38/40A. Immobilized MBP or MBP-Slx5 recombinant proteins were incubated with recombinant GST or GST-tagged Smt3 or GST-Smt3-K30/40A proteins. Immunoblots of inputs and pull-downs were probed with antibodies against MBP or GST, as indicated. *C*, yeast two-hybrid assays revealed decreased interactions between Smt3-K38/40A and Ufd1. *S. cerevisiae* strains expressing Ufd1 or Cdc48 fused to the Gal4 AD, and strains expressing three tandem repeats of SMT3ΔGG or Smt3-K38/40AΔGG fused to DBD, were generated. Ten-fold serial dilutions of indicated strains were spotted on the indicated media. Protein interactions result in activation of the ADE2 reporter gene and growth on plates lacking adenine (-Ade). SIM, SUMO-interacting motif; MBP, maltose-binding protein; Ufd1, ubiquitin fusion degradation protein 1.

Lysines 38 and 40 reside within a surface of Smt3 that mediates noncovalent interactions with other proteins through SIM binding. To evaluate the effect of K38/40A mutations on interactions between Smt3 and SIM-containing proteins, we performed two-hybrid assays using strains expressing a Smt3-Gal4 DNA-activation domain fusion protein and a Slx5-Gal4 DNA-binding domain fusion protein. Slx5 is a STUbL that contains tandem SIMs with high affinity for polymeric SUMO chains (30). To enhance Smt3–Slx5 interactions, WT Smt3 or the Smt3-K38/40A mutant were expressed as linear trimeric fusion proteins bound to the Gal4 DNA-activation domain. The C-terminal diglycine motif was deleted from each Smt3 protein (ΔGG) to prevent cleavage of the fusion protein and covalent attachment to other proteins. We detected robust interactions between WT Smt3 and Slx5 fusion proteins, as indicated by an activation of the *ADE2* reporter gene and growth on plates lacking adenine (Fig. 4*A*). In contrast, this interaction was severely reduced in cells expressing the Smt3-K38/40A fusion protein. Negligible growth was detected when either WT Smt3 or Smt3-K38/40A fusion proteins were expressed in the presence of Slx5 fusion proteins containing SIM mutations that prevent SUMO–SIM interaction (Fig. 4*A*).

To further investigate effects of the K38/40A mutation on SIM binding, we also performed *in vitro*–binding assays using purified recombinant proteins. Slx5 was expressed as a maltose-binding protein (MBP) fusion protein, purified from bacteria, and immobilized on amylose beads. Beads containing MBP-Slx5 or MBP alone were incubated with bacterially expressed and purified GST, GST-tagged WT Smt3, or the Smt3-K38/40A mutant. Following incubation and washes, bound proteins were analyzed by SDS-PAGE and immunoblotting. This analysis revealed a >5-fold reduction in binding between the Smt3-K38/40A mutant and Slx5 compared to binding between WT Smt3 and Slx5 (Fig. 4*B*). Binding specificity was confirmed by undetectable interactions between Smt3 and MBP alone. Thus, both *in vivo* and *in vitro* assays demonstrate that the K38/40A mutation in Smt3 reduces affinity for the SIMs in Slx5.

To further evaluate effects of the K38/40A mutation on Smt3-SIM binding and their potential implications in NES-GFP-Ura3-CL1 degradation, we also assessed interactions between WT and Smt3-K38/40A with the Cdc48 cofactor, ubiquitin fusion degradation protein 1 (Ufd1). Ufd1 contains a C-terminal SIM that mediates interactions with sumoylated substrates (31) and the Cdc48-Npl4-Ufd1 complex is required for efficient degradation of Ura3-CL1 in yeast (21). We performed two-hybrid assays using *S. cerevisiae* strains expressing Cdc48 or Ufd1 fused to Gal4 DNA-activation domain and strains expressing three tandem repeats of Smt3ΔGG or Smt3-K38/40AΔGG fused to Gal4 DNA-binding domain. Interaction was detected between Ufd1 and WT Smt3 fusion proteins, and this interaction was severely reduced in strains expressing Smt3-K38/40A fusions (Fig. 4*C*). We did not observe direct interactions between Smt3 and Cdc48.

### The Smt3-K38/40A mutant exhibits near normal rates of conjugation and deconjugation

**Figure 5:**
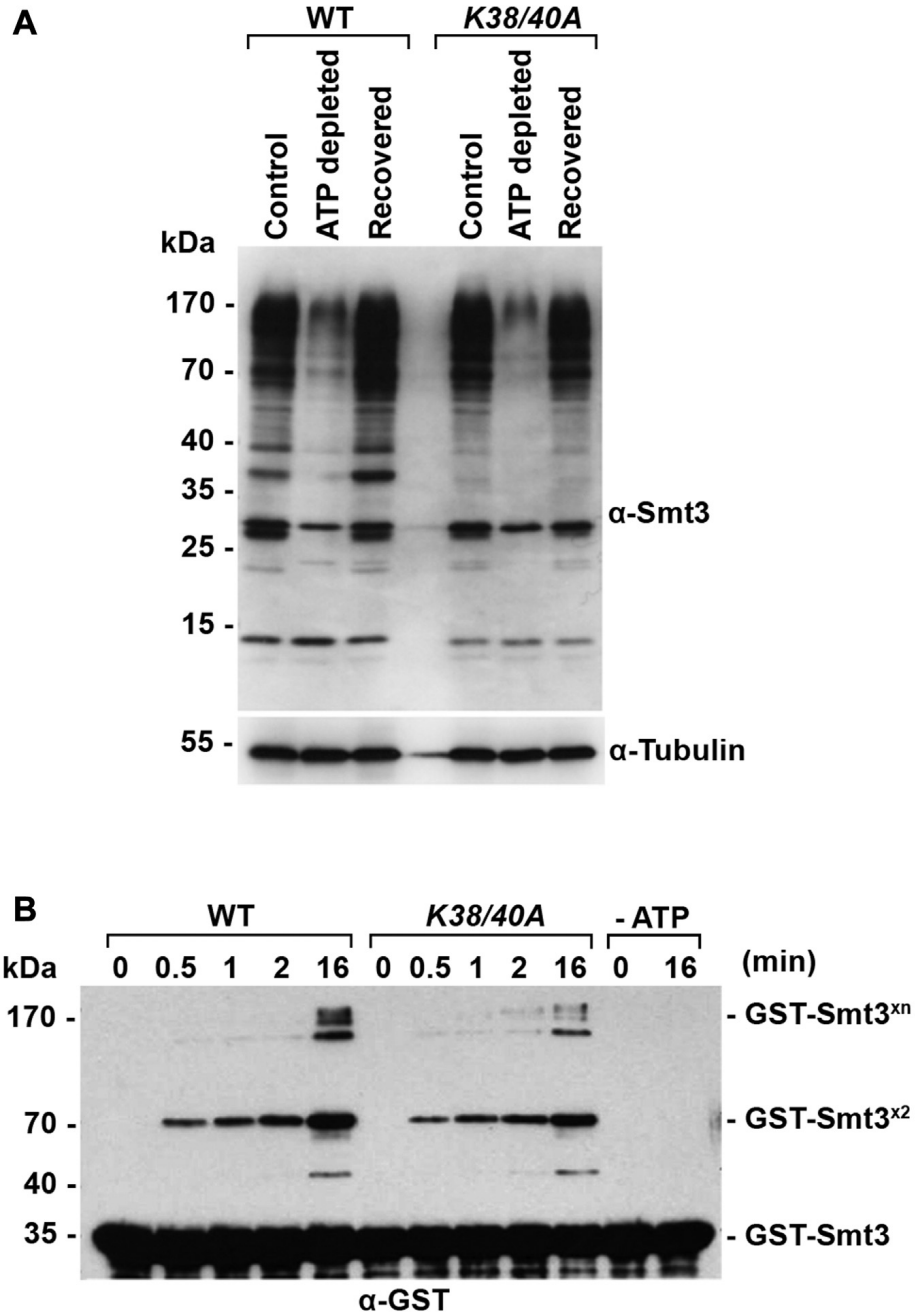
**The Smt3-K38/40A mutant protein is not defective in conjugation or deconjugation.***A*, *in vivo* analysis of deconjugation and conjugation follows ATP depletion and restoration. Cultures at mid-log phase were grown in normal medium (control) or medium containing sodium azide and 2-deoxyglucose (ATP depleted) for 10 min. Cells were then allowed to recover for 10 min in normal medium (Recovered). Total cell lysates were immunoblotted with antibodies as indicated. *B*, *in vitro* analysis of WT and Smt3-K38/40A protein conjugation. Purified recombinant GST-tagged WT or Smt3-K38/40A proteins were incubated with purified E1 activating and E2 conjugating enzymes in the presence of ATP for the indicated times. WT protein and enzymes were also incubated in the absence of ATP as a negative control. Smt3 chain formation was detected by immunoblot analysis using anti-GST antibodies.

Because SUMO pathway enzymes also contain potentially important SIMs, including the E1-activating enzyme, E3 ligases and isopeptidases (32), we investigated possible effects of the K38/40A mutation on Smt3 conjugation and deconjugation using both *in vivo* and *in vitro* assays. First, we evaluated conjugation and deconjugation kinetics of WT Smt3 and the K38/40A mutant *in vivo* by culturing mid-log phase cells in the presence of sodium azide and 2-deoxyglucose for 10 min to deplete ATP and then allowing them to recover for 10 min in normal medium. Because SUMO conjugation is ATP-dependent and deconjugation is ATP-independent, ATP depletion will lead to a rapid loss of high molecular mass conjugates, which can be rapidly recovered when cells are returned to normal culture medium. This assay showed a nearly identical loss of high molecular mass signals in WT and *Smt3-K38/40A* mutant strains upon ATP-depletion and a near identical recovery of conjugates upon return to normal culture medium (Fig. 5*A*). However, although conjugated and deconjugated with similar efficiencies, the pattern of modified proteins detected in WT and *Smt3-K38/40A* mutant strains showed notable differences. These included the absence of prominent bands at 37 and 40 kDa in the mutant strain.

To further assess effects of the K38/40A mutation on conjugation kinetics, we also performed *in vitro* assays using purified recombinant E1-activating and E2-conjugating enzymes. E1 and E2 enzymes were incubated with either GST-tagged WT Smt3 or the Smt3-K38/40A mutant in the presence or absence of ATP. Following incubation for varying lengths of time, the production of polymeric Smt3 chains was evaluated by SDS-PAGE and immunoblotting. Consistent with *in vivo* results, this analysis revealed comparable rates of dimeric Smt3 chain formation (Fig. 5*B*). Thus, based on both *in vitro* and *in vivo* analyses, we conclude that Smt3-K38/40A is defective in SIM binding and that functional consequences of this defect likely involve interactions with effector proteins acting downstream of conjugation.

### SUMO1 regulates GFP-CL1 degradation in mammalian cells

**Figure 6:**
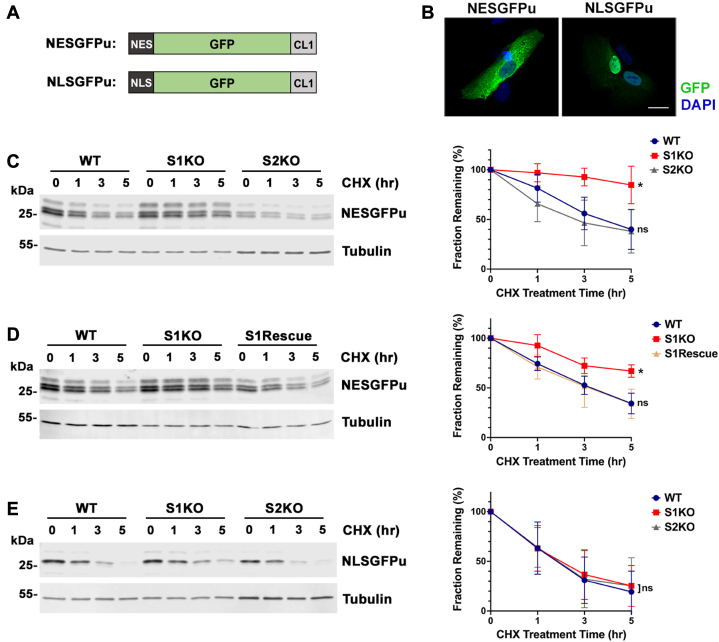
**SUMO1 is uniquely required for efficient degradation of a cytosolic reporter protein in mammalian cells.***A*, diagrams of CL1 degron-containing reporter proteins. *B*, WT cells were transfected with NESGFPu and NLSGFPu expression constructs. Localization to the cytoplasm and the nucleus, respectively, was verified by immunofluorescence microscopy. Scale bar represents 20 μm. *C*, degradation of NESGFPu was defective specifically in SUMO1 KO (S1KO) cells. Cells were transfected with the NESGFPu construct, and 100 μg/ml cycloheximide (CHX) was administered 24 h posttransfection. NESGFPu intensities at indicated time points were detected using anti-GFP antibodies. Tubulin was detected as a loading control. Signal intensities were measured using ImageJ and graphs were generated using the Prism software. GFP/Tubulin ratios at time 0 were set to 100%, and protein half-life was estimated based on the curve where remaining GFPu levels were reduced to 50% of starting levels. Error bars represent the SD from four biological replicates. Statistical analysis was performed on the last time point (5 h) using an ordinary one-way ANOVA test. *p* values reflect differences in protein levels relative to NESGFPu levels in WT cells. ∗*p* = 0.029, ns *p* =0.892. *D*, the degradation defect in SUMO1 KO cells can be rescued by stable reintroduction of SUMO1 expression. Quantification and statistical analysis was performed as in (*C*). Error bars represent the SD from three biological replicates. ∗*p* = 0.031, ns, *p* =0.972. *E*, degradation rates of the nuclear-localized NLSGFPu reporter protein were comparable between WT and SUMO1 and SUMO2 KO cells (S1KO and S2KO). Quantification and statistical analysis was performed as in (*C*). Error bars represent the SD from three biological replicates. ns, *p* =0.987. SUMO, small ubiquitin-related modifier; NES, nuclear export signal; NLS, nuclear localization signal.

**Figure 7:**
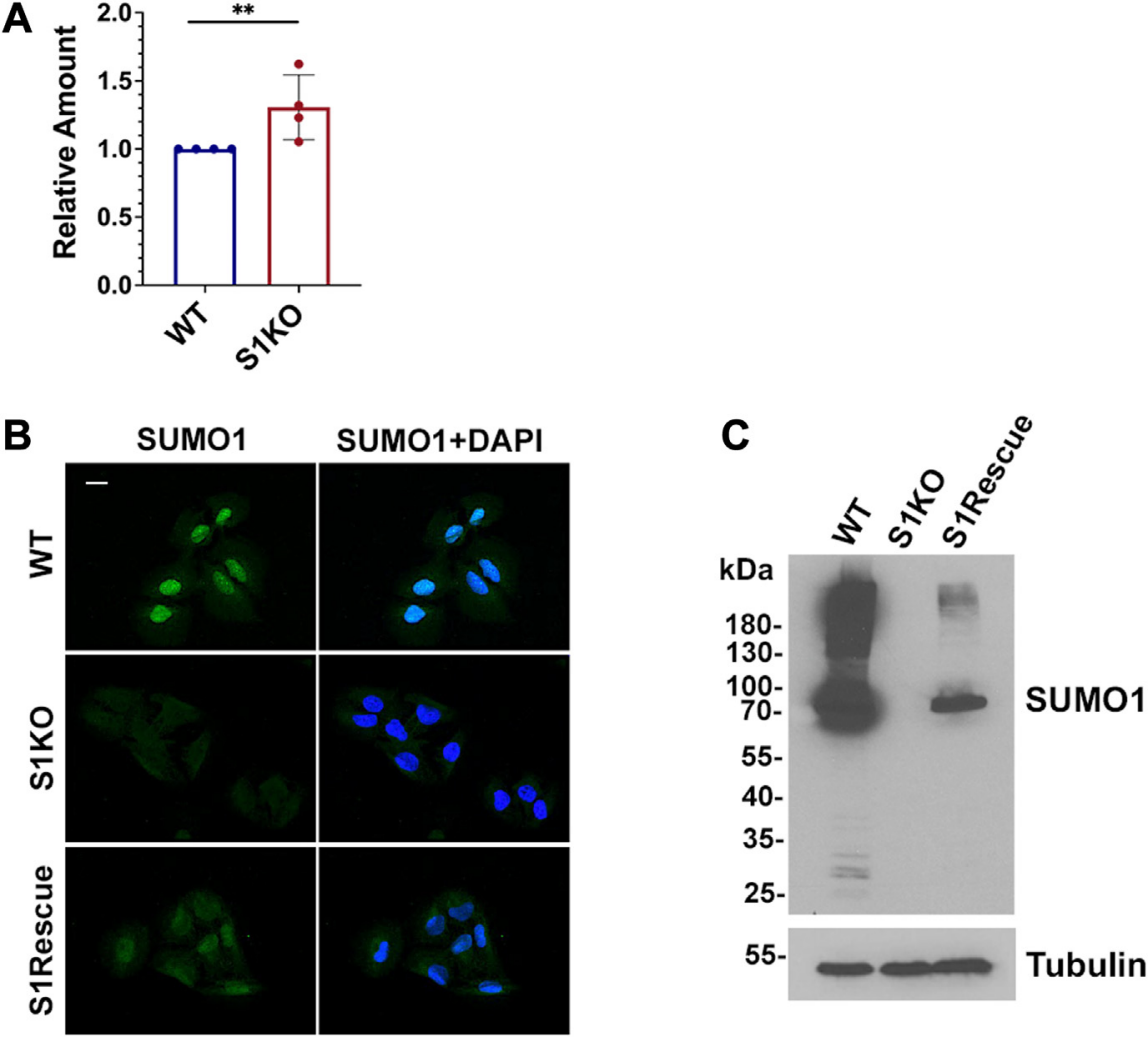
**Analysis of steady state levels of NESGFPu and verification of a SUMO1 rescue cell line.***A*, SUMO1 KO cells have higher steady state NESGFPu protein levels than WT cells. WT and SUMO1 KO cells were transfected with the NESGFPu expression construct, and immunoblot analysis of whole cell lysates was performed with anti-GFP antibodies. Tubulin was used as loading control. Signal intensities were measured using ImageJ and steady-state abundance of NESGFPu in WT cells was set to 1. Statistical analysis was performed using a one sample t and Wilcoxon test. Error bar represents the SD from four biological replicates. ∗∗*p* = 0.002. *B*, stable SUMO1 re-expression in a SUMO1 rescue (S1Rescue) cell line was verified by immunofluorescence microscopy (scale bar represents 20 μm) and (*C*) immunoblot analysis. NES, nuclear export signal; SUMO, small ubiquitin-related modifier.

A recent study revealed that the pathway for turnover of CL1-containing reporter proteins by ER-resident ubiquitylation machinery is conserved in mammalian cells (24). To explore whether a role for sumoylation in cytoplasmic PQC was also conserved across species, we took advantage of recently described human U2OS SUMO1 and SUMO2 KO cell lines (33). We transfected WT U2OS and SUMO KO cells with a mammalian construct coding for the cytosolic, CL1-degron–containing reporter protein, NES-GFP-CL1 (referred to hereafter as NESGFPu) (Fig. 6, *A* and *B*) (34). Cells were treated for varying lengths of time with cycloheximide to inhibit new protein synthesis, and protein turnover was evaluated by immunoblot analysis (Fig. 6*C*). Quantification showed a significant stabilization of NESGFPu in SUMO1 KO cells (t_1/2_ >5 h) compared to WT and SUMO2 KO cells (t_1/2_ ≈ 3 h). Consistent with this increased stability, we detected an increase in the steady state expression level of NESGFPu in SUMO1 KO compared to WT cells (Fig. 7*A*). Importantly, stable re-expression of SUMO1 fully rescued the cytosolic degradation defect in SUMO1 KO cells (Figs. 6*D* and 7, *B* and *C*). To further explore whether the degradation defect is also restricted to the cytoplasm in mammalian cells as observed in yeast, we transfected cells with a construct coding for a nuclear-localized reporter protein, NLSGFPu (Fig. 6*B*), and observed no difference in stability between WT and SUMO KO cells (Fig. 6*E*). Collectively, our results show that sumoylation plays a conserved role in facilitating degradation of cytoplasmic PQC reporter proteins containing the CL1 degron and that this function is unique to the SUMO1 paralogue in human cells.

### SUMO1 regulates NESGFPu degradation upstream of ubiquitylation and proteasome activity

**Figure 8:**
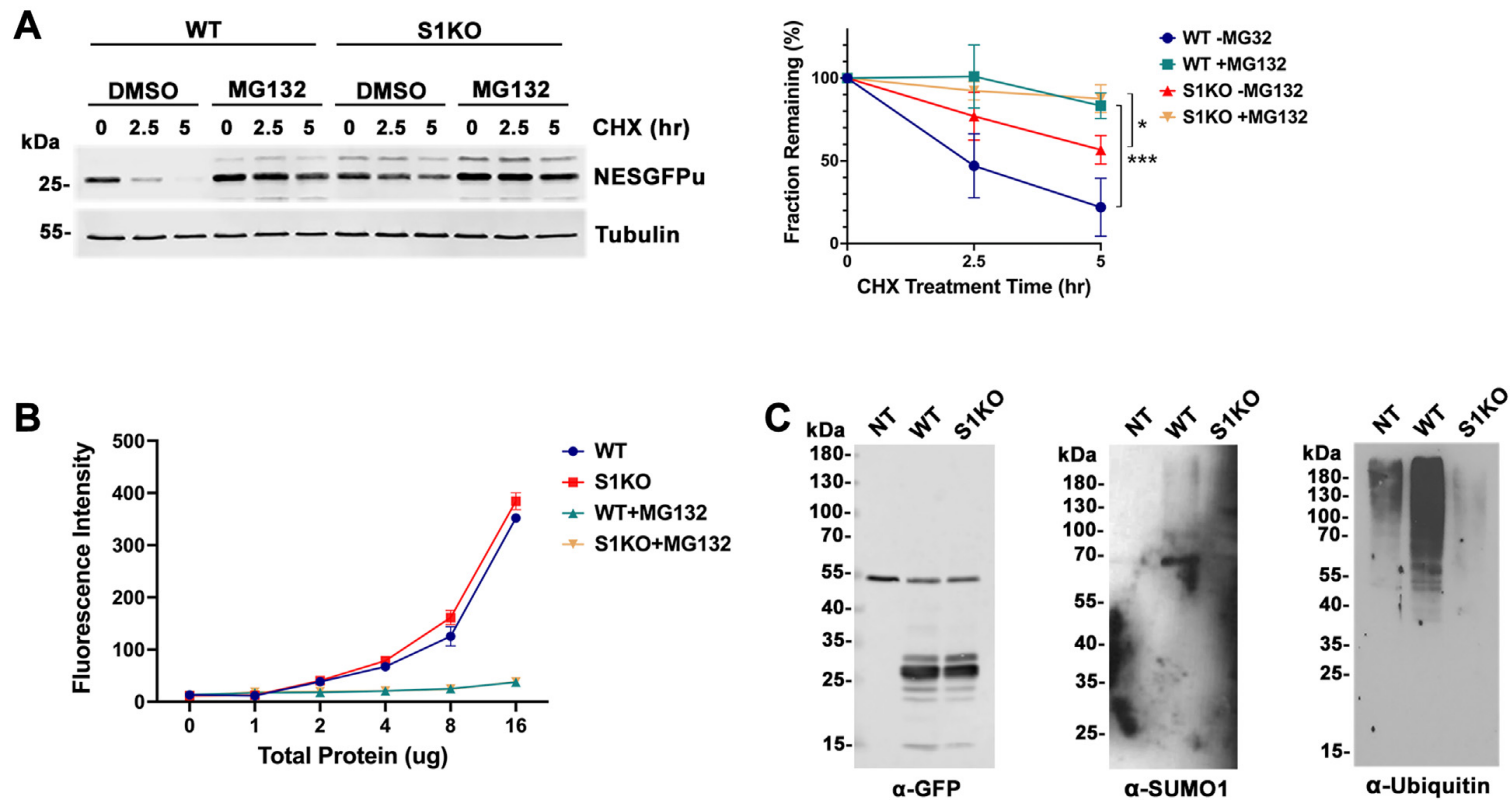
**SUMO1 regulates NESGFPu degradation upstream of ubiquitylation and proteasome activity.***A*, NESGFPu is degraded by the proteasome in U2OS cells. WT and SUMO1 KO cells were transfected with the NESGFPu expression construct and subsequently treated with 20 μM MG132 for 2 h prior to coincubation with cycloheximide. Degradation of NESGFPu was followed for 5 h. Signal intensity was measured and statistical analysis was performed as in Figure 6. Error bars represent the SD from three biological replicates. *p* values reflect differences in protein levels in WT and SUMO1 KO cells treated with MG132 relative to respective DMSO control. ∗∗∗*p* = 0.001, ∗*p* = 0.030. *B*, *in vitro* proteasome activity assays showed no quantifiable difference in proteasomal activity between WT and SUMO1 KO cells. The cleavage-activated fluorescence proteasome substrate, Suc-LLVY-AMC, was incubated with indicated amounts of total protein extracts from WT and SUMO1 KO cells and fluorescence intensity was measured and plotted. *C*, immunopurification revealed ubiquitin and SUMO1 modification of NESGFPu. NESGFPu was purified from WT and SUMO1 KO cells under denaturing conditions using anti-GFP magnetic beads. Covalent conjugation with ubiquitin and SUMO1 was detected using antibodies against each protein. NT, nontransfected control; NES, nuclear export signal; SUMO, small ubiquitin-related modifier.

Stability of GFPu reporter proteins has been widely used as a readout for proteasome activity in mammalian cells (34, 35, 36). To evaluate whether the observed cytoplasmic PQC defect in SUMO1 KO cells was due to reduced proteasome activity, we first evaluated the turnover of NESGFPu in U2OS cells treated with the proteasome inhibitor MG132. As expected, NESGFPu turnover was inhibited by MG132 treatment in both WT and SUMO1 KO cells (Fig. 8*A*). We then measured relative proteasome activity in WT and SUMO1 KO cells using an *in vitro* proteasome activity assay. This analysis revealed no detectable difference in proteasome activity between WT and SUMO1 KO cells (Fig. 8*B*), indicating that SUMO1 affects misfolded protein degradation upstream of the proteasome.

We next evaluated the alternative hypothesis that SUMO1 regulates NESGFPu turnover through direct modification of the substrate by performing anti-GFP immunopurification and immunoblotting. NESGFPu was immunopurified from WT and SUMO1 KO cells using anti-GFP beads under protein denaturing conditions. Pull-down fractions were then analyzed by immunoblot with anti-GFP antibodies to verify immunopurification and with anti-SUMO1 and anti-ubiquitin antibodies to detect covalent posttranslational modifications. We detected high molecular mass SUMO1-reactive signals in pull-downs of NESGFPu from WT cells, but not in pull-downs from SUMO1 KO cells or nontransfected WT control cells (Fig. 8*C*). Of note, high molecular mass conjugates were not detected in the anti-GFP blot, which we attribute to an inability to detect low steady state levels of modification. Additionally, we detected a smear of high molecular mass ubiquitin conjugates in NESGFPu pull-downs from WT cells (Fig. 8*C*). Notably, levels of these ubiquitin conjugates were severely reduced in the pull-down fraction from SUMO1 KO cells, indicating that SUMO1 modification functions upstream of ubiquitylation.

**Figure 9:**
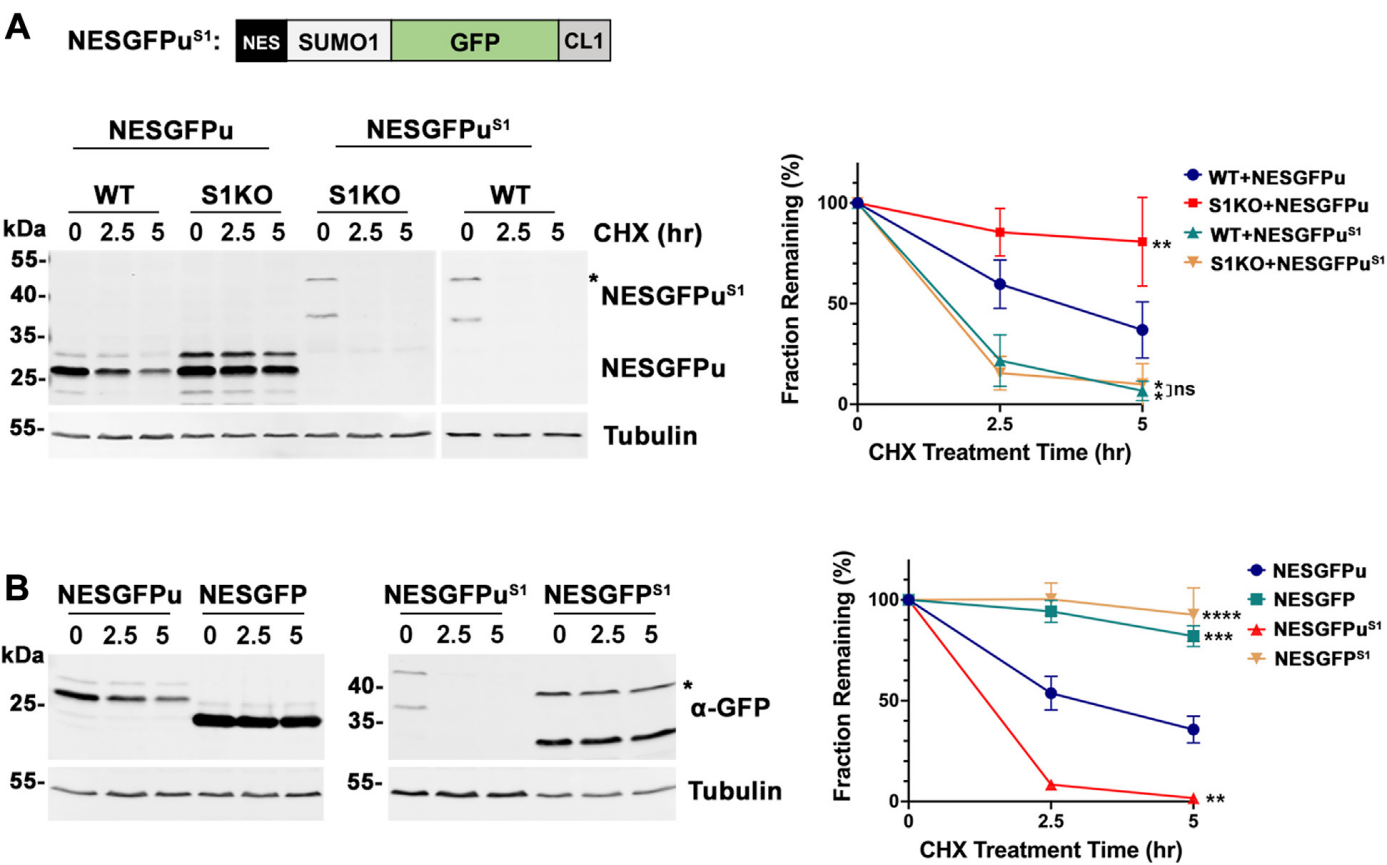
**SUMO1 directly and selectively regulates degradation of degron-containing substrate.***A*, direct SUMO1 fusion to NESGFPu rescued the degradation defect in SUMO1 KO cells. WT and SUMO1 KO cells were transfected with indicated constructs. Asterisk indicates the predicted full-length SUMO1 fusion protein (NESGFPu^S1^). Signal intensities of top bands were measured and quantified as in Figure 6. Error bars represent the SD from four biological replicates. Statistical analysis was performed on the last time point (5 h) using an ordinary one-way ANOVA test. *p* values reflect differences in protein levels compared to NESGFPu levels in WT cells. ∗∗*p* = 0.004, ∗*p* = 0.032 (WT) and 0.039 (S1KO), ns, *p* = 0.752. *B*, SUMO1 selectively regulates degradation of degron-containing substrates. WT cells were transfected with indicated constructs. Asterisk indicates predicted size of full-length SUMO1 fusion proteins. Protein stability was quantified, and statistical analysis was performed as in Figure 6. Error bars represent the SD from three biological replicates. Error bars for NESGFPu^S1^ are not visible due to their short lengths compared to the symbol size. *p* values reflect differences in protein levels compared to NESGFPu levels in WT cells. ∗∗∗∗*p* < 0.0001, ∗∗∗*p* = 0.0003, ∗∗*p* = 0.0015. NES, nuclear export signal; SUMO, small ubiquitin-related modifier.

To further investigate possible direct effects of SUMO1 conjugation on NESGFPu stability, we transfected cells with a construct coding for a NES-SUMO1-GFPu fusion protein (NESGFPu^S1^). Consistent with direct SUMO1 conjugation functioning to facilitate protein turnover, stability assays showed that the half-life of NESGFPu^S1^ was reduced to ∼80 min compared to >5 h for NESGFPu alone in S1KO cells (Fig. 9*A*). Similarly, reduced protein half-lives were observed in WT and SUMO1 KO cell lines, indicating that the degradation defect in SUMO1 KO cells was fully rescued. The steady state expression levels of NESGFPu^S1^ were also reduced in both cell lines, consistent with their increased turnover. We also analyzed the effect of fusing SUMO1 to NESGFP lacking the CL1 degron. NESGFP had a half-life >5 h and this was not enhanced by fusion with SUMO1 (Fig. 9*B*). Collectively, these observations demonstrate that SUMO1 selectively facilitates the turnover of unstable protein substrates and functions upstream of ubiquitylation and proteasome activity.

### RNF4 depletion does not affect NESGFPu degradation

**Figure 10:**
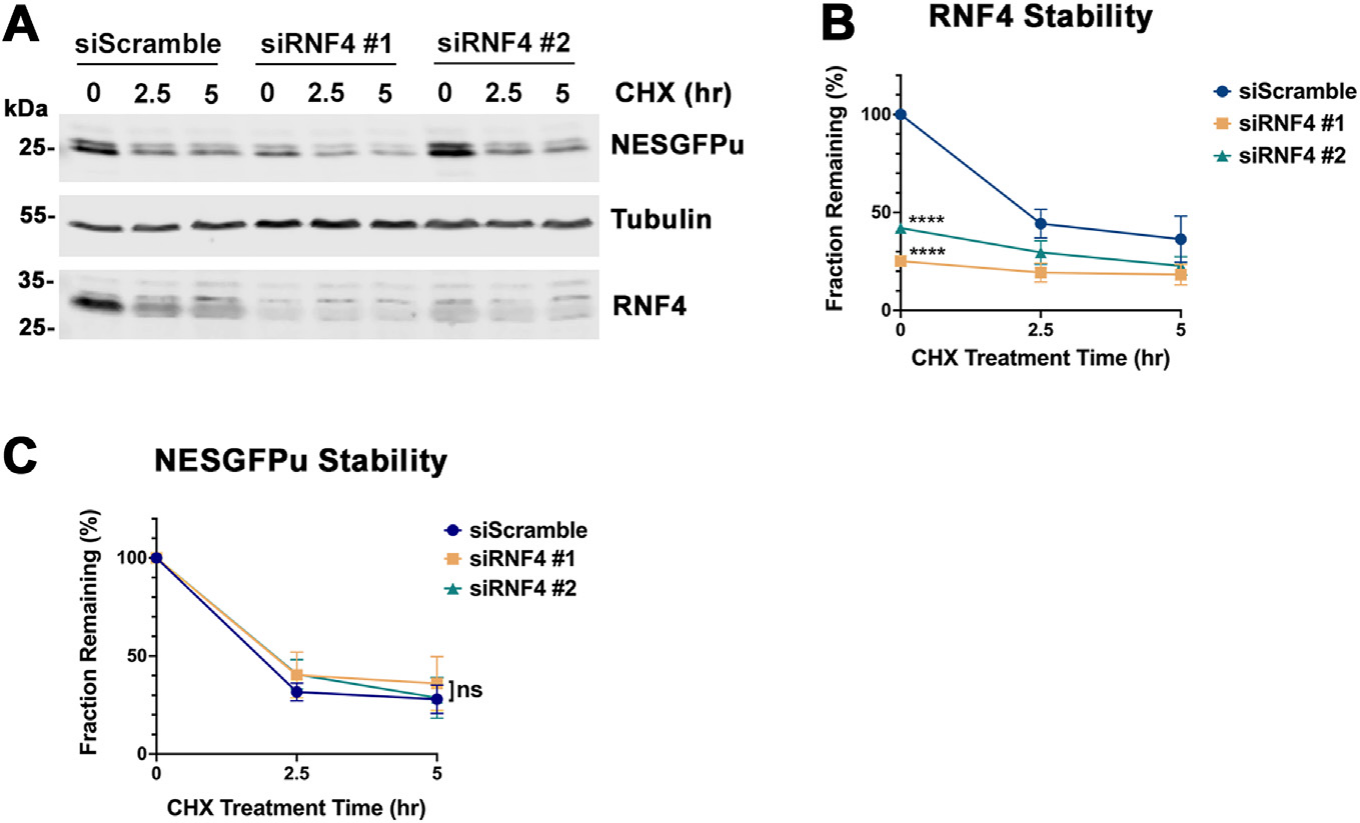
**RNF4 depletion does not affect NESGFPu degradation.***A*, cells were cotransfected with NESGFPu plasmid and either a scrambled siRNA control (siScramble) or RNF4-specific siRNA oligos (siRNF4 #1 and siRNF4 #2). Cycloheximide was administrated 48 h posttransfection and cell lysates were prepared and analyzed with antibodies against the indicated proteins at the indicated time points. *B*, quantification of RNF4 turnover and RNAi-mediated depletion. RNF4 signal intensities at each time point were measured using ImageJ, normalized to tubulin, and plotted. The RNF4 signal detected in siScramble-treated cells at time 0 cycloheximide was set to 100%. Error bars represent SDs from three biological replicates. *p* values reflect changes in RNF4 protein levels at time 0 compared to siScramble-treated cells. ∗∗∗∗*p* < 0.0001. Statistical analysis was performed using an ordinary one-way ANOVA test using the Prism software. *C*, NESGFPu stability was not affected by RNF4 depletion. NESGFPu signal intensities were measured and analyzed as in Figure 6. Error bars represent SDs from three biological replicates. Statistical analysis was performed using an ordinary one-way ANOVA test using the Prism software. *p* values reflect changes in NESGFPu stability compared to siScramble-transfected cells. ns *p* = 0.783 (siRNF4 #1) and 0.942 (siRNF4 #2). NES, nuclear export signal.

SUMO-modified proteins can be recognized and ubiquitylated by STUbLs, leading to their proteasomal degradation. The STUbL protein RNF4 was previously shown to mediate degradation of misfolded nuclear proteins modified by polymeric SUMO2/3 chains, but its involvement in cytosolic PQC is unclear (7, 19). To test whether RNF4 is involved in NESGFPu degradation, we knocked down endogenous RNF4 expression in WT U2OS cells using two independent siRNA oligos and evaluated the effects on NESGFPu stability. The knockdown efficiency was ∼75% for oligo #1 and ∼60% for oligo #2 (Fig. 10, *A* and *B*). Using cycloheximide treatment, we found that the degradation rate of NESGFPu was not significantly different between control treated cells and cells treated with individual RNF4-specific siRNA oligos (Fig. 10*C*). Importantly, this was true between 0 to 2.5 h cycloheximide treatment, where RNF4 protein levels were initially depleted in RNAi-treated cells compared to control cells. Notably, RNF4 was also depleted with cycloheximide treatment in siScramble-treated control cells (t_1/2_ ∼ 2.5 h), consistent with its known short half-life (37) and further demonstrating that it is not a crucial mediator of NESGFPu instability. Collectively, our results reveal that SUMO1 regulates cytosolic PQC through a mechanism that is independent of RNF4.

### SUMO1 facilitates NESGFPu degradation by promoting substrate solubility

**Figure 11:**
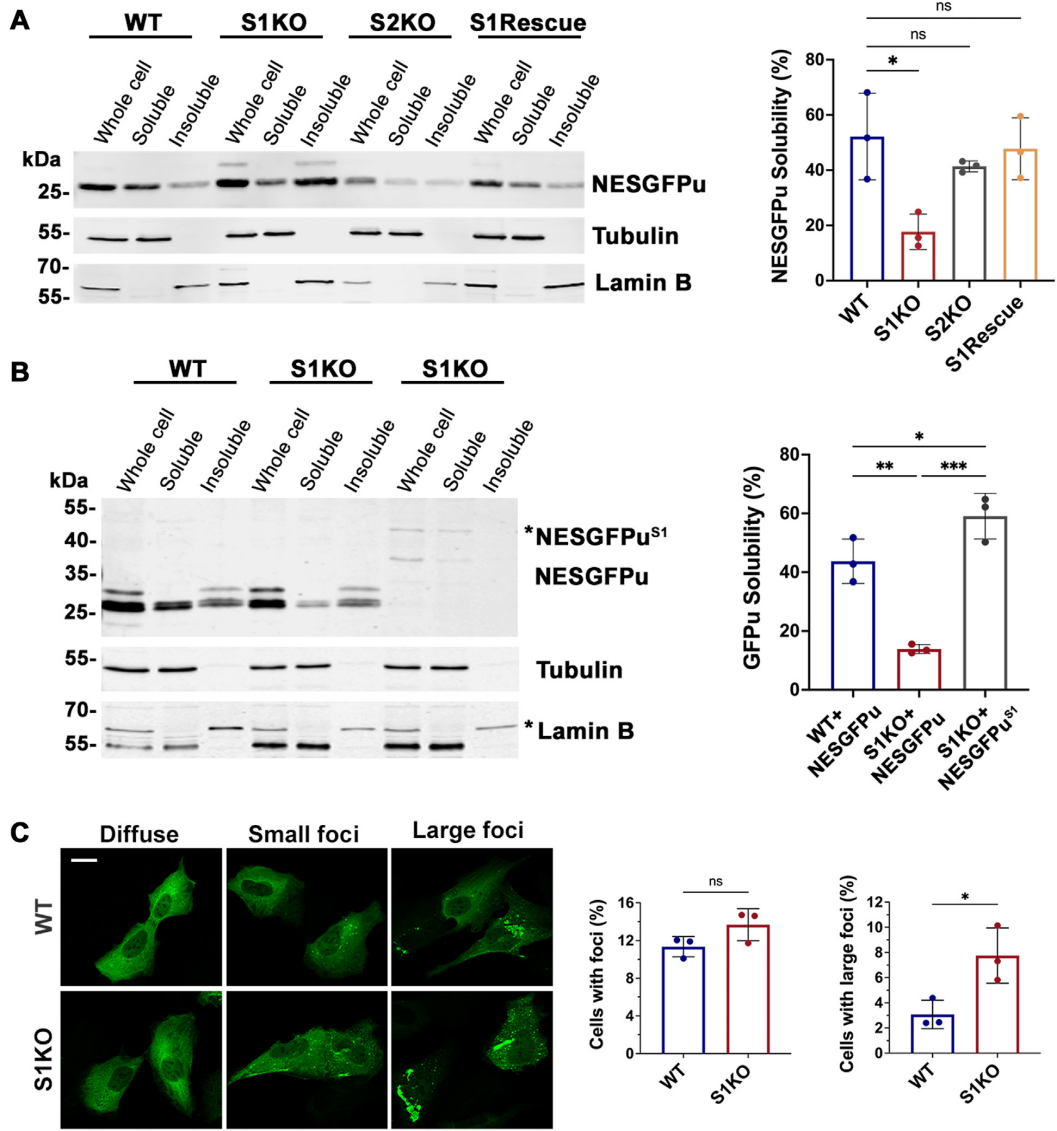
**SUMO1 regulates NESGFPu degradation by promoting solubility.***A*, cellular fractionation assays revealed decreased solubility of NESGFPu in SUMO1 KO cells. The indicated NESGFPu-transfected cell lines were lysed in buffer containing 0.5% deoxycholate, and soluble and insoluble fractions were isolated by centrifugation and analyzed by immunoblotting. Tubulin and lamin B were detected as controls for soluble and insoluble fractions, respectively. Relative NESGFPu solubility was calculated as a ratio of soluble fraction/whole cell fraction signal intensities after normalizing to respective controls. Error bars represent SDs from three biological replicates. Statistical analysis was performed using an ordinary one-way ANOVA test using the Prism software. *p* values reflect changes in protein solubility relative to NESGFPu solubility in WT cells. ∗*p* = 0.019, ns *p* = 0.544 (S2KO), and 0.714 (S1Rescue). *B*, direct fusion of SUMO1 restores solubility of NESGFPu in SUMO1 KO cells. WT and SUMO1 KO cells were transfected with NESGFPu or NESGFPu^S1^-expressing constructs. Fractionation and quantitative analysis of protein solubility were performed as in A. p values reflect changes in protein solubility relative to NESGFPu solubility in WT cells. ∗∗∗*p* = 0.0004, ∗∗*p* = 0.0023, ∗*p* = 0.0246. *C*, SUMO1 KO cells contain more large NESGFPu foci than WT cells. WT and SUMO1 KO cells were transfected with the NESGFPu expression construct, and NESGFPu was detected by immunofluorescence microscopy using anti-GFP antibodies. Scale bar represents 20 μm. Total foci-containing cells and percentage of cells that contain large foci were quantified. Error bar represents the SD from three biological replicates, and >1000 cells were quantified in each replicate. Statistical analysis was performed using an unpaired *t* test. ∗*p* = 0.030, ns *p* = 0.114. NES, nuclear export signal; SUMO, small ubiquitin-related modifier.

Monomeric SUMOs have the potential to promote protein solubility and prevent aggregation (38, 39, 40, 41). To test the hypothesis that SUMO1 facilitates NESGFPu turnover by promoting solubility, we performed cellular fractionation studies. Cells were lysed in the presence of 0.5% deoxycholate, and soluble and insoluble protein fractions were isolated by differential centrifugation. Immunoblot analysis and quantification of relative protein levels in each fraction revealed an over two-fold decrease in NESGFPu solubility specifically in SUMO1 KO cells compared to WT cells (Fig. 11*A*). Like the degradation defect, decreased solubility of NESGFPu was fully rescued by SUMO1 re-expression as well as direct fusion to SUMO1 (Fig. 11, *A* and *B*). We also performed immunofluorescence microscopy to detect potential NESGFPu aggregate formation in the cells. Although primarily diffuse throughout the cytoplasm, NESGFPu formed visible foci in a small fraction of WT and SUMO1 KO cells (11.35% in WT and 13.68% in S1KO), with SUMO1 KO cells containing more large cytosolic foci than WT cells (Fig. 11*C*). The relatively small increase in SUMO1 KO cells with visible large foci suggests that there is not a direct correlation with decreased NESGFPu solubility. Collectively, our results suggest that SUMO1 facilitates cytosolic misfolded protein degradation by maintaining substrate solubility upstream of ubiquitylation and proteasomal degradation.

### The VCP/p97 AAA-ATPase is required for NESGFPu degradation in mammalian cells

**Figure 12:**
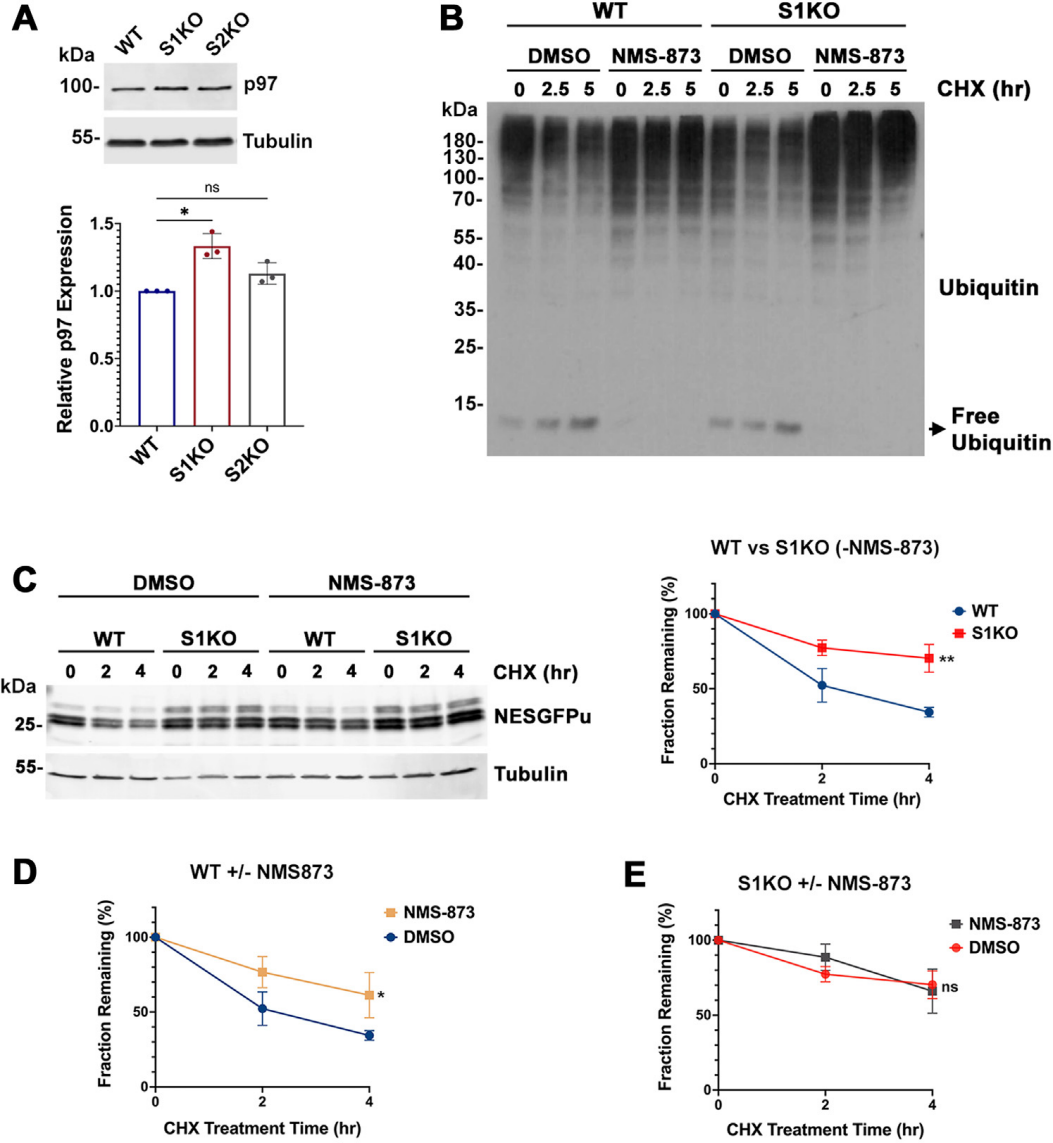
**VCP/p97 is required for efficient degradation of NESGFPu.***A*, anti-p97 immunoblotting showed minor changes in p97 expression levels in SUMO KO cells. Signal intensities were measured using ImageJ, and p97 expression levels in WT cells were set to 1. Statistical analysis was performed using a Kruskal-Wallis test with the Prism software, ∗*p* = 0.019, ns *p* = 0.517. Error bars represent the SD from three biological replicates. *B*, anti-ubiquitin immunoblotting showed increased ubiquitylation levels in NMS-873–treated cells, consistent with VCP/p97 inhibition. *C*, VCP/p97 inhibition by NMS-873 led to defective NESGFPu turnover. Cells were transfected with the NESGFPu expression construct and cultured for 24 h. Cells were then treated with 10 μM NMS-873 or DMSO for 2 h prior to coincubation with 100 μg/ml cycloheximide for the indicated lengths of time. NESGFPu degradation was assessed by immunoblot as in Figure 6. NESGFPu turnover rate was decreased in WT cells treated with NMS-873 compared to DMSO-treated control (*D*), while p97 inhibition by NMS-873 treatment did not affect NESGFPu degradation in S1KO cells (*E*). Error bars represent the SD from three biological replicates. *p* values were calculated using an unpaired *t* test to reflect changes in protein stability based on the last time point (4 h). ∗∗*p* = 0.003, ∗*p* = 0.039, ns *p* = 0.689. NES, nuclear export signal; SUMO, small ubiquitin-related modifier.

The VCP/p97 AAA-ATPase participates in Ura3-CL1 degradation in yeast (21), but its involvement in the degradation pathway in human cells is uncharacterized. VCP/p97 is modified preferentially by SUMO1, and sumoylation is suggested to modulate p97 hexamer assembly and binding to cofactors (42, 43). Furthermore, defects in sumoylation of VCP/p97 attenuate degradation of an ERAD substrate, TCRɑ-GFP (42). To investigate a possible role for VCP/p97 in NESGFPu degradation in human cells, we first quantified VCP/p97 expression levels in WT and SUMO KO cells by immunoblotting. This analysis revealed a slight increase in p97 expression in the SUMO1 KO cells compared to WT cells (Fig. 12*A*). We next treated WT U2OS cells with the selective VCP/p97 inhibitor, NMS-873 (44), followed by measurement of NESGFPu turnover using cycloheximide treatment and immunoblotting. This analysis revealed an increase in NESGFPu stability similar to that observed in SUMO1 KO cells (Fig. 12, *C* and *D*). We also treated SUMO1 KO cells with NMS-873 and observed no additive effect on NESGFPu degradation (Fig. 12*E*), suggesting that VCP/p97 functions in the same pathway as SUMO1. Successful VCP/p97 inhibition by NMS-873 treatment was verified by the detection of expected increases in global ubiquitylation levels using immunoblotting (Fig. 12*B*). Our findings demonstrate that the role for VCP/p97 in turnover of CL1-substrates is conserved from yeast to human.

## Discussion

**Figure 13:**
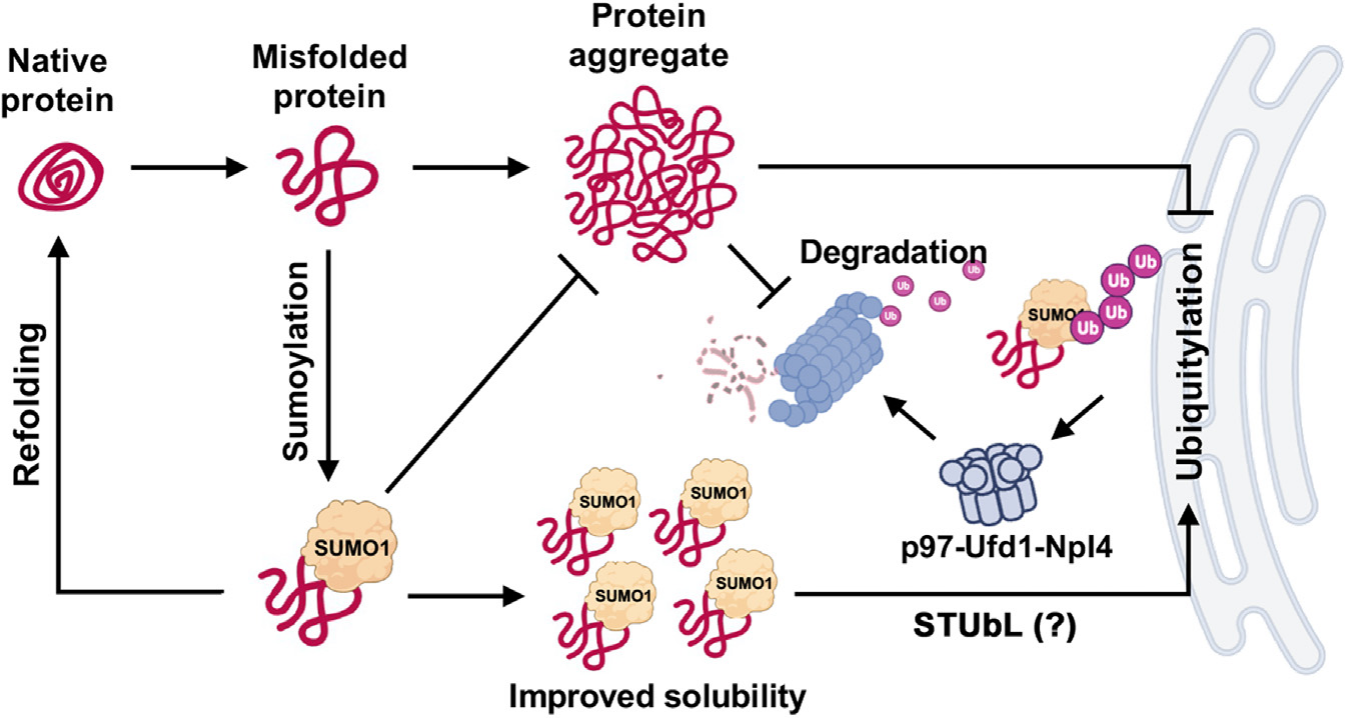
**Proposed model for SUMO1-dependent cytosolic PQC**. Presented is a simplified model whereby SUMO1 modification of misfolded proteins maintains solubility to prevent aggregation and promote targeting to downstream factors required for proteasome degradation. In addition to having a direct effect on protein solubility, SUMO1 may also facilitate interactions with SIM-containing proteins within the pathway, including STUbLs, ER-associated ubiquitylation factors, and the VCP/p97 disaggregase complex. PQC, protein quality control; SUMO, small ubiquitin-related modifier; SIM, SUMO-interacting motif; STUbL, SUMO-targeted ubiquitin ligase.

Mechanisms that direct quality control of soluble, cytosolic misfolded proteins are not well understood. Previously, it has been shown that the ubiquitylation machinery localized on the cytosolic face of the ER serves as a platform for degradation of at least a class of soluble, cytosolic misfolded proteins (20, 21, 24). This pathway was first identified using model protein substrates containing a short, hydrophobic degradation signal known as the CL1-degron, and it remains one of the few cytosolic PQC pathways characterized to date. In the present study, we demonstrate that sumoylation regulates degradation of CL1-degron–containing cytosolic model substrates in yeast and humans. Using human U2OS SUMO KO cell lines, we found that degradation of the CL1-tagged substrate, NESGFPu, is specifically dependent on the SUMO1 paralogue. Knockdown of RNF4 revealed that SUMO1 promotes cytosolic misfolded protein degradation through a mechanism distinct from the previously described nuclear PQC pathway that is dependent on RNF4 and SUMO2/3 polymeric chains (7, 45). Based on our current observations, we favor a simplified model (Fig. 13) whereby SUMO1 (Smt3 in yeast) promotes clearance of cytosolic misfolded proteins by enhancing substrate solubility. We hypothesize that SUMO1 achieves this function through intramolecular interactions with exposed hydrophobic residues within misfolded proteins that serve as SIMs, thereby promoting solubility and preventing aggregation. This is consistent with our findings from immunoprecipitation and proteasome activity assays indicating that SUMO1 regulates misfolded protein turnover by direct conjugation and functions upstream of ubiquitylation and proteasomal degradation. In addition to its role in promoting solubility, it cannot be excluded that SUMO1 also facilitates interactions with SIM-containing proteins involved in ubiquitylation and proteasome targeting, including STUbLs and the p97-Ufd1-Npl4 disaggregase complex, as discussed in the following sections. Our findings that the yeast Smt3-K38/40A mutant has reduced affinities for SIMs in Slx5 and Ufd1 are consistent with this possibility, but additional functional studies are required.

### SUMO1 in cytosolic PQC: passive solubilizer or active adaptor?

Sumoylation is a dynamic process that is upregulated in response to a variety of cellular stresses (46). Increased global SUMO2/3 conjugation upon heat shock, for example, acts as a rapid protective response to proteotoxic stress (47). Our findings suggest that SUMO1 conjugation may also function to protect against misfolded proteins under conditions of proteotoxic stress. This is supported by our finding that the yeast *Smt3-K38/40A* strain was hypersensitive to treatment with amino acid analogs AZC and canavanine, both of which induce protein misfolding. This is also supported by findings that SUMO1 conjugation is upregulated in multiple tissues in response to hibernation-induced ischemia and that this upregulation is protective under ischemic conditions in cultured neuronal cells (48). Surprisingly, however, the yeast *Smt3-K38/40A* strain showed comparable growth properties when cultured under heat stress conditions at 39 °C (Fig. 1*D*). This may be explained by the finding that heat stress activates pathways not fully activated by amino acid analogs (49) and that these additional pathways may be redundant with SUMO-dependent pathways. Further studies are needed to explore this seemingly paradoxical observation.

Our finding that SUMO1 functions upstream of ubiquitylation and degradation, by promoting NESGFPu solubility, suggests a direct and primary role in protein clearance. The exact molecular mechanism by which SUMO1 improves substrate solubility is unclear and requires further investigation. We propose that hydrophobic residues exposed by protein misfolding may be recognized and bound by SUMO1 in a fashion similar to SIM binding, as the canonical SIM itself is composed of a stretch of hydrophobic amino acids (V/I-X-V/I-V/I) (18). In this scenario, conjugated SUMO1 would interact intramolecularly with exposed hydrophobic residues, thereby shielding them from the aqueous cytosol and increasing solubility. This model is supported by our observation that the Smt3-K38/40A mutant protein is defective in SIM-binding but not conjugation or deconjugation (Figs. 4 and 5). It is also consistent with findings that a SUMO1-derived peptide that binds SIMs in ɑ-synuclein suppresses aggregation and cytotoxicity (50).

In addition to promoting solubility, SUMO1 may also direct misfolded substrates, through SUMO-SIM interactions, to downstream degradation machinery. In yeast, ER-localized Ssa1 (Hsp70) and Ydj1 (Hsp40) chaperones were proposed to recognize Ura3-CL1 and direct it to ER-resident ubiquitin E2 (Ubc6, Ubc7, Cue1) and E3 (Doa10) enzymes (21). In humans, degradation of an mCherry-CL1 substrate requires nonredundant activities of E3 ligases MARCH10 (Doa10 homolog) and TRC8, E2 enzymes UBE2G2 (ortholog of Ubc7) and UBE2J2, and the ubiquitin-binding protein AUP1 (ortholog of Ubc7 cofactor Cue1) (24). SUMO1 may function as an adapter that facilitates interactions with these factors. In the current study, we show that RNF4 depletion does not affect SUMO1-dependent cytosolic protein degradation, which is consistent with the specificity of RNF4 for polymeric SUMO2/3 chains and its nuclear localization (19, 44). However, whether MARCH10 or TRC8 function as STUbLs, or whether other STUbLs apart from RNF4 are involved in the pathway, remains to be explored. Regardless of its exact function(s), we anticipate that the role for SUMO1 in cytosolic PQC may be a general pathway in the cell to counteract proteomic stress not limited to the Doa10/MARCH6-mediated degradation pathway described in the current study.

### VCP/p97 in cytosolic PQC: SUMO1 regulated or regulator?

We showed that the AAA-ATPase, VCP/p97, functions in the same pathway as SUMO1 to ensure efficient NESGFPu degradation in mammalian cells. p97 forms a complex with substrate-recruiting cofactors Ufd1 and Npl4, which has been frequently shown to be involved in ERAD and chromatin-associated processes (51, 52, 53). Ufd1 and Npl4 contain both ubiquitin interaction motifs (within the NZF domain of Npl4 and the UT3 domain of Ufd1) and also SIMs (in the Ufd1 C-terminus) (43). Thus, p97-Ufd1-Npl4 acts on both ubiquitin and SUMO-modified substrates, specifically at sites of DNA damage and centromeres in the nucleus (31, 53, 54, 55). It is therefore possible that efficient recognition of cytosolic PQC substrates by the p97-Ufd1-Npl4 complex and their delivery to the proteasome require both ubiquitin and SUMO recognition. Consistent with this, yeast Smt3K38/40A mutant shows decreased interaction with Ufd1 (Fig. 4*C*).

It is also notable that p97 itself is preferentially modified by SUMO1 (43, 56). In addition, sumoylation levels of p97 increase in response to cellular stresses, and defects in p97 sumoylation impair hexamer assembly and attenuate ERAD (42). Thus, whether p97 activity as a segregase and disaggregase is directly regulated by SUMO1 in our proposed SUMO1-dependent PQC pathway is unclear and demands further investigation.

### What are the endogenous substrates for SUMO1-dependent cytosolic PQC?

ER-targeting and degradation of CL1 degron-containing substrates relies on hydrophobicity of the degron (20), which shares some similarities to cellular tail-anchored (TA) proteins. TA proteins feature a single hydrophobic transmembrane domain (TMD) at the very C-terminus and are inherently prone to aggregation (57). A fraction of mistargeted or aberrant TA proteins at the ER and mitochondrial outer membranes goes through the same Doa10-mediated ubiquitylation pathway at the ER cytosolic face as CL1-degron–containing substrates (57, 58, 59). For example, when mistargeted to the outer mitochondrial membranes, the lysosomal TA protein Pex15Δ30 is extracted by the AAA-ATPase and dislocase Msp1 into the cytosol, where it is recognized by the guided entry of TA proteins pathway and targeted to the ER. It is subsequently ubiquitylated by Doa10 with Ubc6, Ubc7, and Cue1, followed by extraction by the Cdc48-Ufd1-Npl4 complex and proteasomal degradation (57, 58, 59). To date, over 50 TA proteins have been identified in yeast, and >400 are predicted in humans (60, 61). Given the structural similarity between CL1 degron-containing substrates and TA proteins, as well as overlaps in their quality control mechanisms, it is reasonable to speculate that SUMO1 may also participate in the quality control of TA proteins. Shielding of TMDs is critical in both TA protein biosynthesis and degradation due to their potential to aggregate. As discussed in the previous section, SUMO1 may function analogously to cellular chaperones by binding and protecting the hydrophobic TMDs and preventing aggregation during TA protein biogenesis and degradation. SUMO1 may also function as an adaptor to facilitate targeting of aberrant TA proteins to designated ubiquitylation machinery. Future studies may be carried out to evaluate how SUMO1 affects quality control of nascent and aberrant TA proteins, especially mistargeted TA proteins that go through the Ubc6-Ubc7-Doa10 ubiquitylation pathway for degradation. Apart from TA proteins, other potential substrates for SUMO1-dependent cytosolic PQC include the numerous neurodegenerative disease-associated proteins known to be SUMO regulated. Sumoylation appears to differentially affect the aggregation of different disease-associated proteins (5, 62). For example, SUMO1 modification of ɑ-synulcein promotes solubility and inhibits fibrillization *in vitro* and *in vivo* (39). However, in the case of various forms of poly-glutaminate proteins such as huntingtin and ataxin-1, which are predominantly nuclear, SUMO1 modification seems to have a deleterious effect and leads to increased cytotoxicity of the proteins (63, 64, 65). These seemingly contradictory observations may be due to distinct outcomes of conjugation by SUMO1 or SUMO2/3 at distinct sumoylation sites or the specific localization of each pathogenic protein. Targeting the sumoylation pathway has been proposed as a potential therapeutic strategy for neurodegenerative diseases (66), but to do so demands a better understanding of the roles and molecular mechanisms through which SUMO impacts specific pathogenic proteins. In future studies, it would be of interest to explore how SUMO1-dependent cytosolic PQC contributes to the clearance of neurodegenerative disease-associated proteins, especially proteins whose cytosolic localization and aggregation are associated with cytotoxicity.

### Summary

SUMOs are predominantly localized to the nucleus of cultured mammalian cells and a majority of published studies have focused on their nuclear functions. Nonetheless, many functions have also been identified for sumoylation in the cytoplasm, including control of ion channels at the plasma membrane, regulation of G-protein signaling, and control of cytoskeleton assembly and dynamics (67, 68). We now show that SUMO also plays a conserved role in regulating PQC in the cytosol, which expands current understandings of the regulatory territory for sumoylation in cells. Results from our studies have provided a potential mechanism by which SUMO1 regulates quality control of misfolded and pathogenic proteins and offer novel insights into the roles for sumoylation in PQC-associated diseases. SUMO1 KO mice are viable and can be used to further explore the roles and mechanisms of SUMO1 in PQC using disease models and primary cells (69, 70, 71, 72). As indicated, the GFPu reporter protein is widely employed as a readout for proteasome activity and as a tool to characterize novel PQC pathways (21, 34). Our findings therefore also suggest caution when using this substrate to reflect proteasome function under conditions where sumoylation may also be affected, such as under cellular stress conditions.

## Experimental procedures

### Yeast strains and genetic procedures

Yeast transformation, genetic procedures, and cell viability assay were performed according to standard protocols (73, 74). Yeast strains used in this study were listed in Table S1. K38/40A mutant strain (JLY1) and its isogenic WT strain (JLY5) were constructed as described previously (25). Briefly, WT or mutant SMT3 allele was integrated by transforming EarI-digested pRS413-*SMT3*^*K38/40A*^*::LEU2* or pRS413-*SMT3*^*WT*^*::LEU2* plasmid into two micron-less SMT3 shuffle strain (a derivative of S288C, *MATa his3Δ1 leu2Δ0 met15Δ0 lys2Δ0 ura3Δ0 smt3::kanMX* [pRS316, *SMT3 CEN-URA3*]) with leucine selection. Transformants were replica printed and Leu+, Ura-, G418-sensitive and 5-FOA–resistant strains were isolated. For degron stability assays, JLY187 and JLY188 were generated by replacing *SMT3*^*WT*^*::LEU2* in JLY5 with *SMT3*^*WT*^*::URA3* and *SMT3*^*K38/40A*^*::URA3*, respectively.

### Plasmid constructions

Plasmids used in this study were listed in Table S2. pRS413-*SMT3*^*K38/40A*^ plasmid was constructed by site-directed mutagenesis based on pRS413-*SMT3*^*K38A*^ plasmid. The resulting plasmid was sequenced to ensure no extra mutations were introduced. Tandem repeats of *SMT3* alleles lacking diGlycines (*SMT3*^*ΔGG*^*X3* and *SMT3*^*K38/40AΔGG*^*X3*) were synthesized by BioBasic Incorporated (Canada) and cloned into yeast 2 hybrid plasmid pGAD-C1 (a gift from Dr Pamela B. Meluh, JHSOM). SLX5 and its SIM mutant derivative (*slx5 SIM^∗^*) (75) were cloned into yeast two-hybrid plasmid pGBD-C1. For expression of GST-SMT3 and GST-SMT3^K38/40A^, *SMT3* and *SMT3*^*K38/40A*^ were cloned into pGEX-6p-1. *MBP-SLX5* plasmid was a gift from Dr Oliver Kerscher (College of William and Mary). Control plasmid expressing only MBP was reported previously (76). *P*_*GAL*_*-NES-GFP-URA3-CL1* and *P*_*GAL*_*-NES-GFP-URA3-CL1* plasmids were a gift from Dr Susan Michaelis (JHSOM). *P*_*GAL*_*-CPY^∗^-HA* was reported previously (77). Mammalian GFPu plasmids were constructed by cloning sequences of subcellular localization signal (NES: MNELALKLAGLDI; NLS: MPKKKRKVGG) and the CL1 degron (ACKNWFSSLSHFVIHL) into EGFP-C1 plasmid. SUMO1 fusion plasmids were constructed by inserting the SUMO1 CDS sequence (with C-terminal diglycine motif removed) upstream of EGFP.

### Phenotypic growth assays

Growth of *SMT3* mutants was analyzed essentially as described (25). Briefly, cells were grown overnight in synthetic complete media in 96-well plates at 30 °C. Overnight cultures were serial diluted 10-fold and spotted onto the test plates. Plates containing canavanine (SC -Arg +Can) or tunicamycin were described previously (25).

### MBP pull-down assay

MBP-tagged SLX5 and MBP proteins were purified from *Escherichia coli* Rosetta strains using amylose Resin (New England Biolabs) as previously described (74). Recombinant GST, GST-SMT3, or GST-K30/40A mutant Smt3 proteins were purified using Glutathione Sepharose 4B (Sigma) from *E. coli* Rosetta strains. MBP pull-down assay was performed as described (75).

### Smt3 conjugation and deconjugation assays

*In vivo* Smt3 conjugation and deconjugation following ATP depletion and restoration were described previously (25). Briefly, log phase cells were washed with 1× PBS, harvested, then resuspended in 1 ml of ATP depletion solution (10 mM sodium azide, 10 mM 2-deoxyglucose in 1× PBS). Cells were incubated in the ATP depletion solution at 30 °C for 10 min. After ATP depletion, half of the sample was removed and frozen as the “ATP depleted” sample. The remaining cells were washed with 1× PBS, harvested, and resuspended in prewarmed synthetic medium. Cells were allowed to recover at 30 °C for 10 min. Following recovery, the cells were harvested and the pellet was frozen as the “Recovered” sample.

### *In vitro* Smt3 chain assembly assay

*In vitro* SUMO conjugation was analyzed as described (78). Briefly, purified WT or K38/40A Smt3 protein (65 mM) was mixed with E1-activating enzyme (Aos1-Uba2, 70 nM), E2-conjugating enzyme (Ubc9, 55 mM), and 5 mM ATP in SUMO Assay buffer (20 mM Hepes [pH 7.3], 110 mM potassium acetate, 2 mM magnesium acetate, 1XProtease inhibitor, 1 mM DTT) for 0.5, 1, 2, or 16 min. WT protein and enzymes were also incubated in the absences of ATP as a negative control. The reaction was stopped by adding 50 mM EDTA to the reaction. Smt3 chain formation was detected by immunoblot analysis using anti-GST antibody. Antibodies used are listed in Table S3.

### Yeast cycloheximide-chase analysis and immunoblotting

Cycloheximide-chase assays were performed as described previously (21). Briefly, cycloheximide was added to a final concentration of 100 μg/ml to inhibit protein synthesis. At indicated time points, 500 μl of cells were harvested by addition to an equal volume of 2 × azide stop mix (20 mM NaN_3_, 0.5 mg/ml bovine serum albumin) on ice. Cells pellets were frozen at -80 °C until preparation of cell extracts. SDS-PAGE and immunoblotting were performed as described previously (25). Antibodies used are listed in Table S3.

### Yeast live cell immunofluorescence imaging

Log-phase WT or *K38/40A smt3* mutant strains were treated with canavanine (150 mg/ml) or untreated for 2 h. Can1-GFP localization was determined by fluorescence microscopy as previously described (25).

### Mammalian cell culture, transfection, and cycloheximide-chase analysis

U2OS cells were grown at 37 °C, 5% CO2 in Dulbecco's Modified Eagle Medium (Life Technologies, CAT:11965-092) supplemented with 10% fetal bovine serum (Atlanta Biologics, CAT: S11550). For GFPu stability analysis, cells were seeded in 6-well plates and transfected using Lipofectamine 2000 Reagent (Invitrogen, CAT: 11668027) following commercial protocol. Five hundred nanograms NESGFPu or S1-NESGFPu plasmids are transfected into each well and 100 μg/ml cycloheximide (Sigma-Aldrich CAT: 01810) was administered 24 h posttransfection for designated lengths of time.

### Immunoblotting

Cells were lysed with 2× Laemmli buffer (4% SDS, 20% glycerol, 125 mM Tris–Cl, pH 6.8, 10% 2-mercaptoethanol, 0.02% bromophenol blue) and denatured at 95 °C for 5 min. Whole cell lysate was loaded onto 12.5% SDS–polyacrylamide gels and transferred onto polyvinylidene difluoride membrane. Membranes were blocked in 5% milk (in TBS) followed by overnight incubation at 4 °C with primary antibodies (anti-GFP[1:500], anti-Tubulin[1:5000], anti-Lamin B [1:5000], anti-SUMO1 [1:1000], anti-p97 [1:2000]) and incubation at room temperature for 1 h with horseradish peroxidase–conjugated secondary antibodies ([1:10,000] in 5% milk) or IRDye fluorescence secondary antibodies ([1:10,000] in TBS-T). Bound antibodies were visualized by autoradiography following incubation of membranes with Amersham ECL prime Western blotting detection reagent (CAT: 45-002-401) or fluorescence imaging using an Odyssey infrared imager (LiCOR), respectively. Immunoblot signal intensities were quantified using FIJI image processing software (79) (https://imagej.net/learn/) and normalized to corresponding loading controls. For stability assays, protein half-life was determined by normalizing remaining GFPu amounts at each time point to time 0 values. Antibodies used are listed in Table S3.

### Immunoprecipitation using ChromoTek GFP-trap magnetic agarose

Cells were seeded in 6-cm culture dishes and transfected with 1 μg GFPu-expressing construct. Cells were cultured overnight and treated with 20 μM protease inhibitor MG132 (EMD Millipore, CAT: 474787) for 2 h prior to lysis to increase global sumoylation levels. Cells were washed with prewarmed PBS and lysed with 200 μL ice-cold lysis buffer (10 mM Tris–HCL pH7.5, 150 mM NaCl, 0.5 mM EDTA, 0.1% SDS, 1% deoxycholate, 1% Triton X-100, 1 mM DTT) supplied with 20 mM N-Ethylmaleimide and protease inhibitor cocktails. Lysates were cleared by brief sonication and dilute 2:3 with dilution buffer (10 mM Tris–HCL pH7.5, 150 mM NaCl, 0.5 mM EDTA, 1 mM DTT, 20 mM N-Ethylmaleimide + protease inhibitors). Fifty microliters lysate from each sample were saved as in-put fraction and mixed with an equal amount of 2× Laemmli buffer. For anti-GFP pull-down, 80 μl bead slurry was used per sample, and beads were equilibrated 3× with dilution buffer. Five hundred microliters diluted lysate were added to beads and rotated end-over-end for 1 h at 4 °C. Fifty microliters unbound fraction were saved as flow-through and mixed with an equal amount of 2× Laemmli buffer. Beads were then washed 3× with 500 μl–diluted RIPA buffer and 3× with dilution buffer on a magnetic stand to remove unbound proteins. After the final wash, beads were resuspended with 50 μl 2× Laemmli buffer and boiled at 95 °C for 5 min. Samples were subjected to immunoblotting and probed with indicated antibodies. Antibodies used are listed in Table S3.

### Cellular fractionation

Cells were seeded in 6-cm culture dishes and transfected with 1 μg NESGFPu-expressing construct. Cells were lysed 24 h posttransfection with 200 μl fractionation buffer (10 mM Tris–HCL pH7.5, 150 mM NaCl, 0.5 mM EDTA, 1% NP-40m 0.5% deoxycholate, 1 mM DTT) supplied with protease inhibitor cocktail (1 mM PMSF, 2 μg/ml aprotinin, 10 μM leupeptin, add immediately before use) and briefly sonicated. Cleared lysate from each sample was divided into two equal halves as whole cell lysate and for fractionation respectively. For fractionation, lysates were centrifuged at 13,000 rpm for 20 min at 4 °C. Supernatant (soluble fraction) was collected by carefully pipetting, and pellet (insoluble fraction) was resuspended with 100 μl fractionation buffer. Equal amount of 2× Laemmli buffer was added to each fraction and boiled at 95 °C for 5 min. Samples were subjected onto 12.5% SDS–polyacrylamide gels for immunoblotting, and signal intensities were analyzed using the FIJI software. Solubility of NESGFPu substrates was calculated as soluble fraction/whole cell fraction after normalizing to corresponding tubulin-loading controls.

### MG132 treatment and proteasome activity assay

Cells were transfected with NESGFPu plasmids and cultured for 24 h. Cells were then treated with 20 μM MG132 (EMD Millipore, CAT: 474787) for 2 h before administration of 100 μg/ml cycloheximide for designated lengths of time. NESGFPu protein stability was assessed by immunoblotting as previously described. For *in vitro* proteasome activity assay, cells were seeded in 10 cm culture dish and harvested with ice-cold 0.5% NP-40 (in PBS). Lysates were incubated on ice for 10 min and centrifuged at 13,000 rpm, 4 °C, for 15 min to remove debris. Supernatants were collected and protein concentration was determined by BCA assay following standard protocol. Serial dilutions were performed to obtain samples containing 0, 1, 2, 4, 8, 16 μg total proteins. Proteasome activities were detected using a fluorometric proteasome substrate (Suc-Leu-Leu-Val-Tyr-AMC, AdipoGen, CAT: AG-CP3-0016) following established protocol (https://www.abcam.com/ps/products/107/ab107921/documents/Proteasome-Activity-Assay-Kit-protocol-v8g-ab107921%20(website).pdf). Fluorescence output was detected using a fluorometric plate reader.

### RNF4 knockdown

Cells were cotransfected with 500 ng NESGFPu plasmids and 20pmol of indicated siRNA oligos using Lipofectamine 2000 Reagent following commercial protocol. ON-TARGETplus human RNF4 siRNA oligos were purchased from Dharmacon (siRNF4 #1: J-006557-07-0002, siRNF4 #2: J-006557-09-0002). Cells were incubated for 48 h prior to treatment with 100 μg/ml cycloheximide for designated lengths of time. NESGFPu protein stability was assessed by immunoblotting as previously described.

### NMS-873 treatment

For p97 inhibition using NMS-873 (Ape Bio, CAT: B2168), cells were seeded in 6-well plate and transfected with 500 ng NESGFPu. At 24 h posttransfection, cells were treated with 10 μM NMS-873 or dimethyl sulfoxide (DMSO) for 2 h before administration of 100 μg/ml cycloheximide. Cells were then cultured in the presence of NMS-873 and CHX for designated lengths of time before harvesting with 2× Laemmli buffer. Protein stability was analyzed as previously described.

### Immunofluorescence microscopy

Cells were seeded on coverslips in 6-well plate and transfected with 500 ng NESGFPu plasmid. Cells were fixed 24 h posttransfection with 4% formaldehyde in PBS for 20 min followed by permeabilized in 0.25% Triton X-100 in PBS for 20 min. Cells were then incubated with anti-GFP primary antibodies for 1 h, washed in PBS with 0.5% Tween (PBS-T), and incubated with Alexa Fluor secondary antibodies for 40 min. Coverslips were then mounted using Fluoroshield Mounting Medium with 4′,6-diamidino-2-phenylindole (Abcam, CAT: ab104139). All processes were conducted at room temperature. Microscopy images were taken using an upright Zeiss Observer Z1 fluorescence microscope with an Apotome VH optical section grid. Representative images showing NESGFPu proteins subcellular localization and aggregation status in each cell line were taken using a 63 × objective. Antibodies used are listed in Table S3.

## Data availability

All relevant data are contained within this article.

## Supporting information

This article contains supporting information (25, 72, 73, 74).

## Conflict of interest

The authors declare that they have no conflicts of interest with the contents of this article.
