# Supplementary material for: Analysis of a degron-containing reporter protein GFP-CL1 reveals a role for SUMO1 in cytosolic protein quality control
Source: J Biol Chem. 2022 Dec 29;299(2):102851. doi: 10.1016/j.jbc.2022.102851 (PMC9898758; doi:10.1016/j.jbc.2022.102851)
Supplement: Supplemental tables [file mmc1.docx]

**Table S1 Yeast strains used in the study**

| **Strain** | **Genotype** | **Source** |
| --- | --- | --- |
| HNY137 | *MATa his3Δ1 leu2Δ0 met15Δ0 lys2Δ0 ura3Δ0 smt3::kanMX [pRS316, SMT3 CEN-URA3]* | Newman et al., 2017 (25) |
| JLY1 | *MATa his3Δ1 leu2Δ0 met15Δ0 lys2Δ0 ura3Δ0 SMT3^K38/40A^::LEU2* | This study |
| JLY5 | *MATa his3Δ1 leu2Δ0 met15Δ0 lys2Δ0 ura3Δ0 SMT3^WT^::LEU2* | This study |
| JLY187 | *MATa his3Δ1 leu2Δ0 met15Δ0 lys2Δ0 ura3Δ0 SMT3^WT^::URA3* | This study |
| JLY188 | *MATa his3Δ1 leu2Δ0 met15Δ0 lys2Δ0 ura3Δ0 SMT3^K38/40A^::URA3* | This study |
| HNY286 | *MATa trpl-901 leu2-3,112 ura3-52 his3-200 gal4D gal80D LYS2::GAL1-HIS3 GAL2-ADE2 met2::GAL7-lacZ* | Pamela B. Meluh, JHSOM, MD |
| PJ69-4a | *MATa trp1-901 leu2-3,112 ura3-52 his3-200 gal4Δ gal80Δ LYS2::GAL1-HIS3 GAL2-ADE2 met2::GAL7-lacZ* | James et al., 1996 (72) |
| JLY106 | *MATa his3D1 leu2D0 met15D0 ura3D0 CAN1-GFP SMT3^WT^::URA3* | This study |
| JLY107 | *MATa his3D1 leu2D0 met15D0 ura3D0 CAN1-GFP SMT3^K38/40A^::URA3* | This study |

**Table S2 Plasmids used in the study**

| **Yeast plasmids** | **Description** | **Source** |
| --- | --- | --- |
| pJL7 | pRS413-*SMT3^K38/40A^::LEU2* | This study |
| pJL8 | pRS413-*SMT3^WT^::LEU2* | Newman et al., 2017 (25) |
| pJL371 | pRS413-*SMT3^WT^::URA3* | Dr. Pamela B. Meluh, JHSOM, MD |
| pJL372 | pRS413-*SMT3^K38/40A^::URA3* | Dr. Pamela B. Meluh, JHSOM, MD |
| pJL82 | pOBD2-*SMT3^WTΔGG^×3* | This study |
| pJL218 | pOBD2-*SMT3^K38/40AΔGG^×3* | This study |
| pJL504 | pOAD-C1-*SLX5^WT^* | Xie et al., 2007 (73) |
| pJL505 | pOAD-C1-*SLX5^SIM*^* | Xie et al., 2007 (73) |
| pJL506 | *MBP-SLX5* | Oliver Kerscher, College of William and Mary, VA |
| pJL438 | *MBP* | Odeh et al., 2018 (74) |
| pJL247 | pGEX-6p-1-*SMT3^WT^* | This study |
| pJL248 | pGEX-6p-1-*SMT3^K38/40A^* | This study |
| pSM1371 | *4XUPRE-lacZ* | Susan Michaelis, JHSOM, MD |
| pJL90 | pGAD-C1 | Pamela B. Meluh, JHSOM, MD |
| pJL88 | pGBD-C1 | Pamela B. Meluh, JHSOM, MD |
| pSM3106 | pRS425-*P_GAL_-NES-GFP-URA3-CL1* | Susan Michaelis, JHSOM, MD |
| pSM3104 | pRS425-*P_GAL_-NLS-GFP-URA3-CL1* | Susan Michaelis, JHSOM, MD |
| pSM1979 | pRS315-*P_GAL_-CPY*-HA* | Susan Michaelis, JHSOM, MD |
| pJL393 | pGAD-C1-*CDC48* | This study |
| pJL395 | pGAD-C1-*UFD1* | This study |
| pJL222 | pGBD-*SMT3^WTΔGG^×3* | This study |
| pJL220 | pGBD-*SMT3^K38/40AΔGG^×3* | This study |
| **Mammalian plasmids** | **Description** | **Source** |
|  | NES-GFP-CL1 (NESGFPu) | This study |
|  | NES-SUMO1-GFP-CL1  (NESGFPu^S1^) | This study |
|  | NLS-GFP-CL1 (NLSGFPu) | This study |
|  | NES-GFP (NESGFP) | This study |
|  | NES-SUMO1-GFP (NESGFP^S1^) | This study |
|  | VCP(wt)-EGFP | Addgene #23971 |

**Table S3 List of antibodies used in this study**

| **Antibody** | **Source** |
| --- | --- |
| SUMO-1 (21C7) | Matunis Lab |
| Smt3 | Pamela B. Meluh, JHSOM, MD |
| Lamin B | Matunis Lab |
| Ubiquitin | NOVUS, NB300-129 |
| GFP | Abcam, ab290 |
| β-Tubulin | Sigma, T2200 |
| RNF4 | Proteintech, 17810-1-AP |
| VCP/p97 | NOVUS, NBP1-81619 |
| HA (Y-11) | Santa Cruz, sc-805 |
| Pgk1 | Santa Cruz, sc-130335 |
| Hexokinase (HK) | Rob Jensen, JHSOM, MD |
| MBP | New England BioLabs, E8032S |
| GST | Santa Cruz, sc-138 |
| HRP-linked anti-mouse IgG | Cell Signaling Technology, #7076 |
| HRP-linked anti-rabbit IgG | Cell Signaling Technology, #7074 |
| IRDye 680RD Goat anti-Mouse IgG | LiCOR, 926-68070 |
| IRDye 800CW Goat anti-Rabbit IgG | LiCOR, 926-32211 |
| Alexa anti-Mouse Fluor 488 | Invitrogen, A-11001 |
